# A clinical-scale BioArtificial Liver, developed for GMP, improved clinical parameters of liver function in porcine liver failure

**DOI:** 10.1038/s41598-017-15021-4

**Published:** 2017-11-06

**Authors:** Clare Selden, James Bundy, Eloy Erro, Eva Puschmann, Malcolm Miller, Delawir Kahn, Humphrey Hodgson, Barry Fuller, Jordi Gonzalez-Molina, Aurelie Le Lay, Stephanie Gibbons, Sherri Chalmers, Sunil Modi, Amy Thomas, Peter Kilbride, Agnes Isaacs, Richard Ginsburg, Helen Ilsley, David Thomson, Galya Chinnery, Ncedile Mankahla, Lizel Loo, C. Wendy Spearman

**Affiliations:** 1 UCL Institute for Liver and Digestive Health, UCL – Royal Free Hospital Campus, UCL Medical School, London, United Kingdom; 2 Dept. of Surgery, UCL Medical School, Royal Free Hospital, London, NW3 2QG UK; 3 Faculty of Health Sciences, University of Cape Town, Groote Schuur Hospital, Cape Town, South Africa

## Abstract

Liver failure, whether arising directly from acute liver failure or from decompensated chronic liver disease is an increasing problem worldwide and results in many deaths. In the UK only 10% of individuals requiring a liver transplant receive one. Thus the need for alternative treatments is paramount. A BioArtificial Liver machine could temporarily replace the functions of the liver, buying time for the patient’s liver to repair and regenerate. We have designed, implemented and tested a clinical-scale BioArtificial Liver machine containing a biomass derived from a hepatoblastoma cell-line cultured as three dimensional organoids, using a fluidised bed bioreactor, together with single-use bioprocessing equipment, with complete control of nutrient provision with feedback BioXpert recipe processes, and yielding good phenotypic liver functions. The methodology has been designed to meet specifications for GMP production, required for manufacture of advanced therapy medicinal products (ATMPs). In a porcine model of severe liver failure, damage was assured in all animals by surgical ischaemia in pigs with human sized livers (1.2–1.6 kg liver weights). The BioArtificial liver (UCLBAL) improved important prognostic clinical liver-related parameters, eg, a significant improvement in coagulation, reduction in vasopressor requirements, improvement in blood pH and in parameters of intracranial pressure (ICP) and oxygenation.

## Introduction

Acute liver failure (ALF) is frequently a catastrophic event as the clinical course is often complicated by multi-organ failure with cerebral oedema being the terminal event. Acute liver failure can occur at any age, is commonly idiosyncratic with no specific therapy and patients present to hospital critically ill. Approximately 15% will recover spontaneously; the remainder fulfil the Kings College transplant criteria and 85% of those are listed on an urgent transplant waiting list. Currently in UK more than 20% of patients die on the waiting list. Liver disease is the only major disease in the UK on the increase, whilst donor organ availability is almost static, thus there is a large unmet medical need for an alternative treatment. Since the liver has the capacity to repair and regenerate given time, a solution is to provide temporary liver function to “buy time” either for complete recovery or to find a suitable donor organ. We have developed a bio-artificial liver machine based on a biomass comprised of human-liver derived cells^1–4^ combined with adsorptive removal of DNA and endotoxin, to be used in an extracorporeal circuit, treating the whole plasma fraction of the patient over several hours. The human liver is 1.2–1.6 kg containing 1–2 × 10^11^ hepatocytes; experimental and clinical evidence demonstrates that patients can survive even if they have only ~30% of liver function^5^. The aim of this study was to develop methodology appropriate to GMP production for a cell therapy to be delivered on the clinical scale, delivering ~70 billion cells and to test it in a severe, non-reversible model of acute liver failure in pigs with liver weights equivalent to those in humans.

## Materials and Methods

### Ethics statement

We confirm that all methods were carried out in accordance with relevant guidelines and regulations. Title of animal ethics approval: Assessment of the efficacy and safety of a BioArtificial liver machine (UCLBAL) on short term survival of pigs with induced liver failure. Animal Ethics Committee, University of Cape Town, South Africa Application No.: 014/011, Date received: 20/02/14, Date approved: 06/05/14. We confirm that all experimental protocols were approved by a named institutional and/or licensing committee.

Title of human ethics approval: Cultures of human liver cells obtained at surgery: 38/2000 Royal Free local research ethics committee. Royal Free Hospital Hampstead NHS Trust, London UK. Date approved 27/02/02.

Statistics were as described in legends to figures, typically Students t test, paired or unpaired as appropriate. For technical reasons, some of the *in vivo* observations were incomplete for all pigs, however, all the available data is presented with “n” numbers. The datasets generated during and/or analysed during the current study are available from the corresponding author on reasonable request.

### Master and Working Cell Banks

Master and Working cell banks (MCB, WCB) of HepG2 cells (ECACC Wiltshire) were produced to GMP and fully tested to regulatory standards, including molecular cell line identity, such that they can subsequently be used in human cell therapy. 2 ml vials of cells were stored in a GMP cryobanking facility (Fisher Bioservices, Stortford, Hertfordshire), and each preparation was derived from a fresh vial from the WCB.

### Monolayer culture

A WCB vial of single cells was thawed from liquid Nitrogen storage and used to seed a monolayer triple flask (500 cm^2^, Thermo Scientific Loughborough, Leicestershire) in antibiotic-free culture media (MEMalpha, PAA, Pasching, Austria) supplemented with Foetal Calf Serum (10%, PAA, Pasching, Austria) and insulin (0.27 IU/mL, Novo Nordisk, Bagsværd, Denmark) and passaged after 4 to 7 days growth. Cells were acclimatised to supplemented antibiotics during the second passage (penicillin/streptomycin (45 u/ml pen, 45 µg/ml strep), Thermo Fisher Scientific: Life Technologies, Paisley, Scotland) and fungizone (1.1 µg/mL, Thermo Fisher Scientific:Life Technologies, Paisley, Scotland), and at the third passage used to seed 7 x Cell Factories (Easy Fill 10, 6320 cm^2^, Thermo Scientific, Loughborough, Leicestershire). Cells (139 × 10^6^ cells in 1.7 L culture media) were used per Cell Factory and grown for 6 days, and then harvested using TrypLE Select (Thermo Fisher Scientific:Life Technologies, Paisley, Scotland) dissociation reagent and pelleted at 289rcf (1150 rpm, Thermo Scientific Multifuge X3-R; 4-head swing out rotor) (500 mL Corning centrifuge tubes). Resuspended cells were counted for viable cell number using an NC-100 Nucleocounter (Chemometec, DK-3450 Allerod, Denmark), and then mixed in suspension with alginate for encapsulation.

### Alginate preparation

Alginate (80 g) (FMC Ltd) was dispersed and hydrated as a 2% solution using an L5M-A Laboratory Mixer (Silverson Ltd, Chesham, Bucks) in 0.15 M NaCl containing HEPES (Thermo Fisher Scientific: Life Technologies, Paisley, Scotland), pH7.4.

Cell encapsulation was achieved at a cell concentration of 2 × 10^6^ cells/ml in 1% alginate, using a custom-designed multinozzle (8-nozzle) Jetcutter^Tm^ (GeniaLab GmbH, Germany).

Briefly, 8 L of alginate/cell solution was mixed with a density modifier^4,6,7^. A cutting disc (60 × 100 µm wires) cut the flow of cell mixture into segments that dropped perpendicularly into a stirred polymerisation bath of 0.204 M CaCl_2_ solution, with pressurised delivery of the cell mixture to the encapsulator. Calibration at a flow rate of 20 ml/min/nozzle was achieved by altering the delivery pressure between 450 and 600mbar. 2.5–3 L spherical alginate beads were collected over a period of 30–35 minutes. Polymerisation was continued for a further 5 minutes and then cell beads were washed in DMEM four times. Finally beads were collected in MEM-alpha for transfer to a 15 cm ID chamber for fluidisation, enabling culture to performance-competence to provide a UCLBAL biocartridge, filled with 3-dimensional cell organoids. For control studies a similar process was used to produce equivalent volumes of alginate beads without cell inclusions.

### Fluidised bed bioreactor using a Single Use Bioreactor (SUB) Bioprocess Container

To enable subsequent clinical use of a cell therapy, GMP processes are essential. All methods described for the preparation of the biomass were developed to be GMP appropriate, and single use components utilised where possible. 2.5 L alginate-encapsulated cells (beads/biomass) in the chamber were attached in a perfusion circuit to a stirred tank bioreactor with single use bioprocess container (SUB) of 100 L (Hyclone/Thermo Scientific, Cramlington, Northumberland) containing 116 L complete culture media^3^, MEMalpha (PAA), supplemented with insulin (0.27 IU/mL, Novo Nordisk) and antibiotics (penicillin/streptomycin (45 u/ml (pen), 45 µg/ml (strep) Thermo Fisher Scientific (Life Technologies), Paisley), and fungizone (1.1 µg/mL, GIBCO). Biomass to media ratio was 1:46. Culture media in the SUB had temperature (37 °C), pH (7.4) and Dissolved Oxygen (DO, 21–35%) measured via probes (Broadley James, Silsoe, Bedfordshire), and controlled via the ezControl system (Applikon, Tewkesbury Gloucestershire) and GMP compliant BioXpert SCADA software.

Media perfusion from the SUB to the base of the chamber (typically 380 mL/min, Watson Marlow peristaltic pump) allowed fluidisation of the beads (1.675-fold bed height). A tubing warmer provided additional temperature control to inflowing medium immediately before the chamber (AstothermR Plus 220/220 S, Stihler Electronic GmbH, Stuttgart, Germany; Astotube cat #IFT30410 dimensions 6.8mm OD).

Gassing in the SUB was enabled by Mass Flow Controllers inbuilt in the ezControl unit, and entered the disposable bag via an open pipe line, thus minimising frothing whilst enabling rapid response.

Background air supply in the SUB was set at 150–250 mL/min, provided by compressor (Jun-air, OF302). CO_2_ and bicarbonate solution were used to maintain pH, oxygen was delivered by a stand-alone oxygen concentrator (Newlife Intensity 10, AirSep Corporation, Urmston Manchester); DO in the SUB started at 21% but escalated automatically to 35% in response to measurements recorded post-chamber in the perfusion circuit.

Programmed bespoke ‘recipes’ in BioXpert allowed the dissolved oxygen (DO) setpoint escalation in the SUB, as well as feedback loops to control nutrient input and safety measures.

Proliferation of the biomass in the fluidised bed bioreactor (FBB) took 12 days, with regular batch media changes in increasing proportions, on days 4/5, 7 and 11. Optimisation runs determined maximum biological function, eg protein synthesis by cell spheroids at this time (performance-competence). Daily media samples for lactate and glucose analysis (Analox GM-7 Micro-Stat analyser), and for synthetic function via AFP (alpha-fetoprotein) ELISA were stored at −20C for future analysis. Bead biomass was sampled throughout to determine cell density via Nucleocounter (NC-100, Chemometec, Allerod, Denmark), and metabolic viability, assessed on a 250 μl sample, via fluorescent staining with 0.0128 mg/mL fluorescein diacetate (FDA: Sigma Aldrich, Poole, Dorset) and 0.0256 mg/mL propidium iodide (PI: Sigma Aldrich, Poole, Dorset) vital dyes (final concentration), and NIS Elements imaging software.

At harvest, the required total biomass was transported to the site of the animal trial (Animal operating theatre/ICU, University of Cape Town, Cape Town, South Africa); beads were transferred to 1750 cm^2^ Corning Roller bottles containing oxygenated perfluorodecalin (PFC; F2 Chemicals, Preston Lancashire) and media containing HEPES, pH7.4 (GIBCO), at an optimised ratio of oxygen carrier, media and beads^8^.

### Oxygenation of the FBB

Data collected by BioXpert were used to determine the oxygen consumption of the fluidised biomass over the process period. Oxygen provision to the biomass in the chamber was determined as a function of pressure of inlet oxygen and media flow rate through the chamber.

### Validation of cell viability method

Since accurate quantitation of viability in 3D constructs is not a routine protocol we developed and validated a new assay. The results using the developed analysis protocol were then compared to cell counts and protein synthesis and secretion. At least five fields of beads were captured for image analysis, typically 100 beads.

To compare living biomass with dead cell beads by image analysis, we initiated cell death deliberately induced as negative control in beads by exposure to 50% (w/v) Me_2_SO for 5, 10 and 15 mins at room temperature, which we have established, produces a controllable lethal insult.

Fluorescein diacetate (FDA) identifies viable cells by diesterase conversion to fluorescein, only occurring in viable cells, not simply cellular components, and Propidium Iodide (PI) binds to nuclei in membrane permeable cells. Alginate encapsulated liver cell spheroids in beads (AELS, 250 μl), washed 2x with 1 ml PBS and re-suspended in 500 µl of PBS were stained with 20 µl PI and 10 µl FDA for 90 seconds at ambient temperature, washed 2x with 1 ml PBS and transferred to a microscope slide. Images were taken with a Nikon TE200 microscope with a Nikon DS-Fi1c camera (with a 0.67x adapter), DS-U2 PC control unit and NIS Elements software. A monochrome set-up was used with a PI excitation filter of 510–560 nm and emission filter of 590 nm and an FDA excitation filter of 465–495 nm and emission filter of 515–555 nm.

The quantification method is based on the linearity of fluorescence sum intensity of either PI or FDA to an increasing cell number (sum intensity = sum of pixel intensity of all selected pixels (grey scale level from 0–255)). To apply the viability formula: “% viability = signal FDA/(signal FDA + signal PI) × 100” an equal number of dead and viable cells must provide the same signal (sum intensity). High threshold was set to the maximum of 255 for both FDA and PI. Final low FDA or PI threshold was taken as the average of 10 images containing between 40–60 beads per image, including two high cell density samples (22 × 10^6^ and 26 × 10^6^ cells/ml of beads), relevant for evaluation of performance competent beads at harvest. No overspill between the green and red filter was observed. Available exposure times for NIS-elements suitable for our application were 100 ms 150 ms, 200 ms, 300 ms, 400 ms, for FDA and 600 ms, 800 ms, 1 and 2 seconds for PI.

FDA set-up (viability > 99%): low threshold was obtained by capturing all pixels with visible cells. Optimum exposure time was determined by signal-to-noise ratio (SNR), given by sum intensity of pixels representing cell versus pixels without cells.

PI set-up (viability = 0%, same alginate-encapsulated liver cell spheroids in beads (AELS) used for FDA set-up were killed with 50% Me_2_SO): exposure time and low threshold set to obtain same average sum intensity as for FDA. This method has shown robust, consistent and equivalent data from viabilities over > 20 runs over more than 24 months.

#### Bioassay for inflammatory response to UCLBAL encapsulated spheroids (biomass)

We assessed 12 cytokines using FACS analysis (BD™ (Oxford Science Park, Oxford) Cytometric Bead Array (CBA) Human Inflammatory Cytokine Kit, Cat No. 551811; and Human Th1/Th2 Cytokine Kit, Cat. No. 550749; Human IL-17A Flex Set, Cat. No. 560383; Human IL-8 Flex Set, Cat. No. 558277; and Human GM-CSF Flex Set, Cat. No. 558335) in response to biomass exposure. Plasma conditioned with biomass (alginate encapsulated liver cell spheroids - AELS), or with empty beads (EABs) was exposed for 24 h to human leukocytes (isolated from volunteer blood with confirmed consent), using LPS as a positive control. Peripheral blood mononuclear cell (PBMCs) proliferation was estimated by tritiated thymidine incorporation after exposure to biomass-conditioned plasma.

#### *In vitro* UCLBAL testing with human plasma

“Performance-competent” biomass (Day 12 FBB harvest) in the FBB bioreactor with 2 to 2.4 L biomass was used to treat both bilirubin-spiked (300 µM) fresh frozen normal plasma (n = 2, 8 L), as a partial mimic of liver failure in order to demonstrate bilirubin conjugation specifically, and liver failure plasma from a patient (n = 1), for 8 hours. 4 L plasma were obtained after apheresis plasma exchange in the case of discarded liver failure plasma from a patient who developed acute liver failure due to rifampicin, used as anti-TB prophylaxis, and subsequently listed as a candidate for liver transplantation. Clinical biochemistry results showed the patient’s plasma to have glucose of 7.1 mmol/L, AFP of 2 kU/L, total bilirubin of 84 umol/L, lactate dehydrogenase of 849 U/L, Alkaline phosphatase of 41 U/L, Alanine amino transferase of 91 U/L and Aspartate aminotransferase of 729 U/L, INR of 5 and Ammonia of >286 umol/L.

Hourly samples were collected to assess bilirubin conjugation, analysed at Great Ormond Street Hospital (paediatric VITROS system). Samples were also analysed for glucose consumption, alpha-fetoprotein (AFP) production (as an example of biomass-specific protein synthesis, detectable in human plasma), lactate dehydrogenase (LDH) activity released into the plasma as a measure of cell damage, Alkaline Phosphatase, Alanine Aminotransferase (ALT) and Aspartate Aminotransferase (AST) levels; standard measures of liver function were analysed in house (Royal Free Hospital Clinical Biochemistry Department).

#### *In vivo* porcine model of acute liver failure treated with BioArtificial liver in the full extracorporeal circuit

30–35 kg White-x-Landrace female pigs were used in two groups: the treated group (Cell-BAL, n = 13) contained active 3-dimensional (3D) HepG2-cell spheroids in beads (Beads) in a fluidised bed in the UCLBAL chamber; control group (Control BAL, n = 15) contained fluidised but empty alginate beads in the chamber, otherwise identical. In addition, one pig with liver failure was treated only with plasma in the BAL circuit, and another pig was connected to the control circuit but was not subjected to liver failure; these latter two were not included in control group data, but served to ensure the system was not of itself detrimental to safety as assessed by physiological stability during anaesthesia.

**Figure 1:**
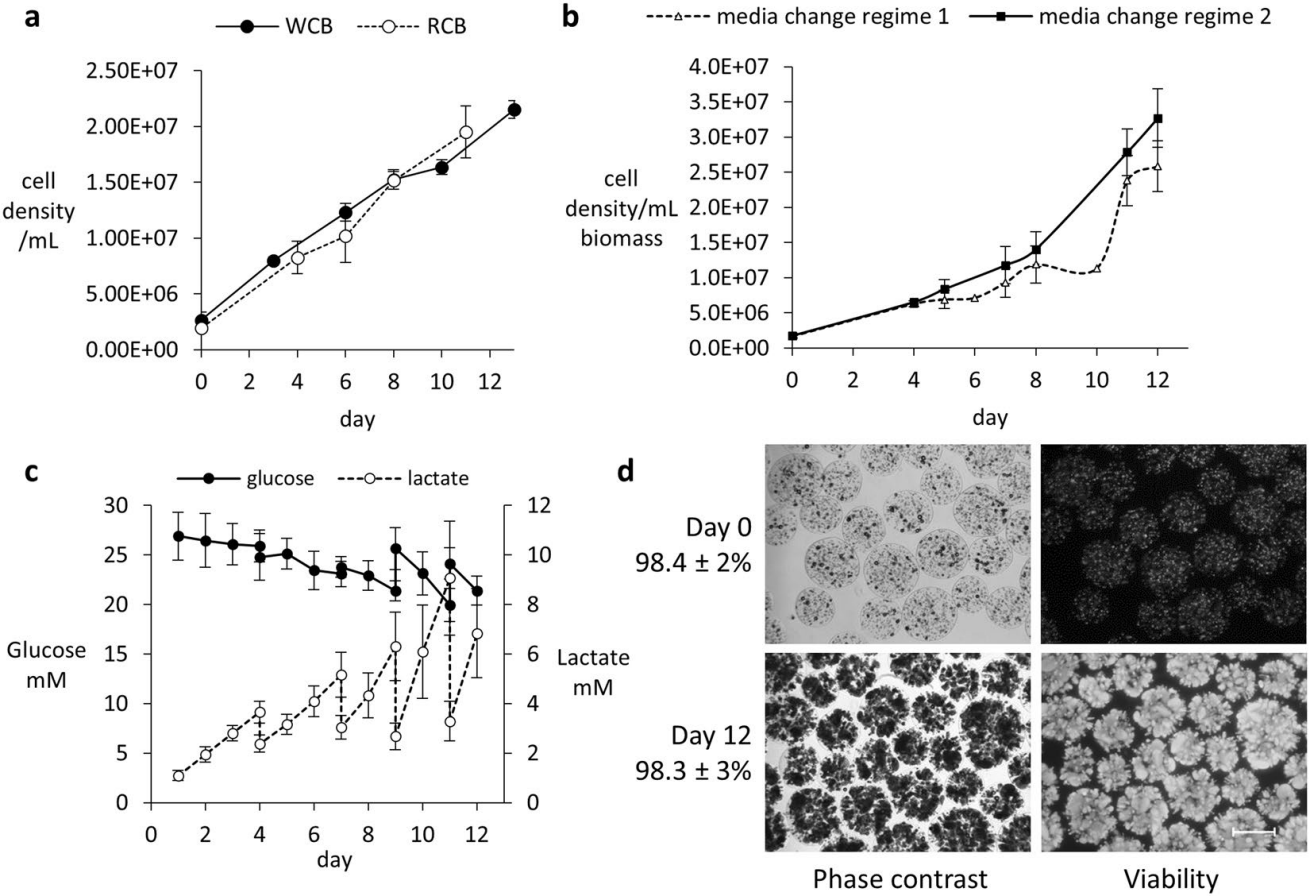
(**a**–**d**) 1A Comparison of GMP working cell bank (WCB) with research cell bank (RCB): cell proliferation. Cell number enumerated by nuclei analysis (Nucleocounter ^TM^) established that proliferation was unchanged between WCB, n = 1, and RCB. (UCL HepG2 research bank)–, n = 3 separate experiments, n = 5 replicates at each time point), in 6-well plates. (b) Media regime during bioprocess: cell proliferation. Cell density in the Fluidised Bed Bioreactor (FBB) was improved with a new media change regime; regime 1(see also Fig. 1c) gave 2.59 × 10^7^ ± 3.6 × 10^6^ cells/mL biomass at day 12 (n = 7, ± SD); regime 2 gave 3.27 × 10^7^ ± 4.18 × 10^6^ cells/mL (n = 14, ±SD); p = 0.0016, Student’s t-test. (**c**). Glucose and lactate monitoring during bioprocess. Using regime 2, (n = 14, ± SD); medium was changed on days 4 (50%), 7 (50/60%), 9 (70%) and 11 (80%); in this way glucose consumption and lactate production were maintained within safe levels and corresponded to replenishment of glucose and removal of lactate. Figure 1d Bead shape, cell proliferation and spheroid viability: image analysis. Left: phase images of encapsulated-HepG2 cell beads during bioprocess. Magnification x4 (Nikon DS-Fi1c camera and NIS Elements software). Right: visualising live cells, stained with fluorescein diacetate (FDA) vital dye, 150 ms exposure time. Average viabilities are shown for each time point; n = 21, ± SD. Propidium iodide staining for dead cells was negligible. Scale bar indicates 500 µm.

For preparation of Cell-BAL, the Biomass was separated from PFC transport medium, culture medium removed, washed copiously with normal saline and resuspended in heparinised normal porcine plasma obtained from the abattoir (1 L) prior to transfer to the fluidised bed chamber in an extracorporeal circuit (Fig. 1 suppl.).

### Ischaemic Acute Liver Failure

#### Anaesthetic regime

Before inducing ischaemic acute liver failure, pigs were fasted overnight. They then received continuous anaesthesia throughout, with induction using doses of zoletil, butorphanol and medetomidine as premed for tracheal intubation, followed by maintenance with isoflurane and/or ketamine, oxygen and nitrous oxide via an endotracheal tube. Fluid management comprised 0.9% saline at 20 ml/kg/hr and boluses of 0.9% saline to maintain stroke volume variation <15% and a CVP of 10 mmHg. Glucose was monitored hourly, and 20 ml boluses of 50% glucose in normal saline was given if glucose levels fell below 4 mmol/L to maintain normoglycaemia at a glucose level of ≥4 mmol/L.

After anaesthetic induction, intracranial pressure (ICP) probes and brain oxygenation catheters were inserted into the brain, with pigs in prone position. Two hours were allowed for physiological brain recovery after the surgical craniotomy; thereafter, ICP and brain oxygenation readings were collected as baseline. Thereafter, the central venous and arterial catheters were inserted prior to initiation of liver failure by surgical ligation of all blood flow to the liver^9^.

In detail, study animals were moved to the supine position, and remained supine throughout, and had vascular catheters inserted into the femoral artery and internal jugular vein to monitor arterial and central venous pressure. The liver was exposed via a midline abdominal incision and the ligamentous attachments were divided. The portal vein and infrahepatic vena cava were dissected, and the hepatic artery and bile duct ligated and divided. The liver was rendered fully ischaemic by ligating the portal vein above a side-to-side portacaval shunt. At completion of surgery a bolus of heparin (3000 units) was administered.

#### Two groups of pigs were treated

The control group were connected to the entire extracorporeal circuit, but the chamber contained empty alginate beads (EABs). The cell-treated BAL group (Cell-BAL) were connected to an identical circuit that contained performance-competent 3-dimensional cell organoids within the alginate beads (AELS) in the chamber. Where n numbers are lower than total numbers in each group, the lower number reflects technical issues and not selective monitoring.

The UCLBAL circuits were connected to a COBE-Spectra centrifugal plasmapheresis instrument (TerumoBCT, Lakewood, Colorado, USA) initially for priming with plasma. The COBE was connected to each animal 20 minutes before connection of BAL at 2.47 ± 0.5 h (n = 29, mean ±SD) following surgical ischaemia via catheters in the external jugular vein with an inlet blood flow of 90–120 ml/min, and a plasma flow of 30–50 ml/min into the primary plasma circuit (slow circuit). The plasma priming the BAL circuit was heparinised during collection of abattoir blood and the circuit maintained with low dose heparin (1–2unit/ml).

Plasma flow rates were fed into the “fast” circuit fluidising the beads in the UCLBAL chamber at a flow rate of 350–500 ml/min to achieve a 2-fold fluidised bed height. The UCLBAL circuit was oxygenated. The “slow” circuit removed BAL-treated plasma continuously for return to the pig via the plasmapheresis instrument at the same flow rate as it was delivered to the BAL, thus ~10% of treated plasma returned and 90% was recirculated at any one time. The functional filters were positioned in the slow circuit to ensure removal of cellular debris, DNA and endotoxin, and particles >0.6 um prior to return to animal (supplementary Figure 1).

#### Intraoperative Monitoring

Blood samples were collected (at −1, 2, 3, 4.5, 6, 7.5, and 9 h from ischaemia (t = 0), and beyond when necessary) and processed for measurement of liver function tests, blood gases and routine haematology (Pathcare Chemical Pathology Service, Cape Town, South Africa). Thromboelastography profiles were obtained using both Haemonetics TEG (Harrow) and Rotem (Queens Meadow, Hartlepool, UK) analyses. Proteins in pig plasma (human albumin, alpha-1-acid glycoprotein, alpha-1-antitrypsin, alpha-1-fetoprotein, fibrinogen and prothrombin) were measured by ELISA; there was no cross reactivity with pig proteins (capture antibodies: from Dako Ltd (Ely, Cambridgeshire): albumin A0001 10 µg/ml, AGP Q0326 1/1000 stock (concentration not specified by supplier), A1at A0012 10 µg/ml; from Abcam plc (Cambridge). AFP ab10071 1 µg/ml, Fibrinogen ab6666/ab10066/ab106659 1 µg/ml, Prothrombin ab22700 1 µg/ml; Horseradish Peroxidase (HRP)-linked antibodies: from Abcam Albumin ab24458–200 0.5 µg/ml, AGP ab34720–10 2.5 µg/ml, AFP ab10072 0.17 µg/ml, A1at ab7635-s 2.5 µg/ml, Fibrinogen ab7539 1 µg/ml /ab106651 1 µg/ml /ab10067 0.7 µg/ml, and Prothrombin from Fisher Scientific PA1-74092, (Bioscience, Nottingham, UK) LS-C39619-200) 2 µg/ml.

Intracranial pressure was measured with a catheter (Camino, 110–4 g Integra Ltd, Andover) inserted into the posterior pole of one hemisphere within the parenchyma at a depth of ~15mm; simultaneously brain oxygenation was measured with a monitor and probe (Licox IT2-EU Complete Brain Tunneling Probe Kit /CC1.P1 Oxygen and Temperature Probe /VK5.2 Parenteral Probe Guide (Integra Life Sciences, Plainsboro, NJ, USA)^10^ inserted into subcortical tissue. Haemodynamics were assessed using PiCCO technology (Pulse Contour Continuous Cardiac Output, Pulsion Medical Systems SE, Germany) via a jugular venous access (Cevox probe, PV2022-32 monitoring ScVO_2_ & PiCCO catheter PV2015L20A.) and arterial catheter (Arrow, Uxbridge, Middlesex, UK: Central Line Venous Catheter set, BR22853), via the femoral artery. Blood gases were measured with ABL80 Flex and ABL8OO Basic blood gas analysers (Radiometer, Crawley, West Sussex, UK).

Control- or Cell-BAL were attached to the test animals for 8 h or until death. Fluid and inotropic support with Noradrenaline were used to maintain haemodynamic stability when required, maintaining a mean systolic pressure >70 mmHg and a cerebral perfusion pressure (CPP) > 60 mm Hg as is used clinically.

## Results and Discussion

### Summary

A clinical sized BioArtificial liver machine (UCLBAL) was prepared and tested in an animal model of ischaemic acute liver failure, using GMP-compatible methodology suitable for use in man as a cell therapy. A biomass of 7.31 × 10^10^ ± 1.7 × 10^10^ viable cells (viability 98.5%, n = 12; variation was not greater than 3%) was transferred at ambient temperature with oxygenation, from site of production to site of use some 9000 kilometres distant, with retained viability, indicating this new therapy can be delivered to the bedside of any hospital worldwide from a central production facility. There was minimal drop in bead viability (98.4 ± 1.0% to 93.0 ±3.2%) when Cell-BAL was connected to pigs with liver failure over course of treatment (6–10 hours from ischaemia; n = 6, ±SD).

Optimal cell spheroid production within the alginate beads relied on close control of nutrients, gases, temperature and waste product removal in fluidised bed format, achieved with recipe control of fermentation process throughout 12 days, using BioXpert software with full CFR pt 11 compliance.

Important parameters of clinical liver failure were improved in the Cell-BAL treated group compared with the control (empty bead) BAL group, most notably coagulation, shown and discussed below.

### GMP master and working cell banks, and FBB bioprocess

The fully tested GMP working cell bank and the initial research cell bank (RCB) performed equally well, despite several changes in protocol to enable GMP production. Figure 1a illustrates proliferation. Protein synthesis and production was also maintained (WCB: 9291 ± 1526 and RCB: 8611 ± 1797 ng AFP/million cells/day

Scaling up for clinical preparations of the BAL biomass required transition from triple flasks to ~6300 cm^2^ cell factories. Hyperflasks (Corning Ltd, 1720 cm^2^) were compared with 10 layer cell factories from Nunc; the latter format was optimal and yielded increased cell number per cm^2^ (Hyperflasks: 1.81 × 10^5^ cells per cm^2^; Cell Factory: 3.57 × 10^5^ ± 5.2 × 10^4^ cells per cm^2^, n = 24). Differences in media to plate-surface ratios and available oxygenation presumably accounted for improvements of cell factories over hyperflasks. For preparation of 2.5 L biomass, 1.56 × 10^10^ ± 2.4 × 10^9^ total viable cells were harvested from Cell Factories (7 × 6320 cm2 per run; n = 24, ±SD) for encapsulation in 1% alginate, as described previously^3^.

Bead diameter, measured microscopically after encapsulation, was 525.8 ± 95 µm, and after 12 days proliferation in the FBB, 537.0 ± 72 µm (mean ±SD, n = 22). Neither bead diameter, nor bead volume, differed significantly during this time, indicating the calcium concentration during production, and culture was not compromised with respect to alginate polymerisation, and was conducive to cell spheroid growth. Since epithelial cells generally have the adherent phenotype the internal structure of the alginate was sufficient to enable normal cell growth and function. Moreover, we have previously demonstrated extracellular matrix synthesis and deposition in the HepG2 cell spheroids within alginate beads, not seen in monolayer culture^11^. The ~2-fold scale up of biomass from our previous study, to human scale required an altered media change regime to achieve optimal proliferation over the shortest time. By changing a day 5 media replenishment of 25% to 50% on day 4, day 12 harvested biomass improved from 2.59 × 10^7^ ± 3.6 × 10^6^ cells/mL (n = 7, ±SD) to 3.27 × 10^7^ ± 4.2 × 10^6^ cells/mL (n = 14, ±SD), p = 0.0016, representing a 26% improvement in production efficiency. This is an important consideration when designing optimum process for later industrial scale up. The average viability of biomass at harvest (d12) was 98.3% ± 3%, SD, n = 21 (Fig. 1d).

A combination of media change regime ensured a constant glucose level above 15 mM for cell nutrition, whilst lactate levels were maintained below 10 mM, which is an important parameter since high lactate concentrations are detrimental to alginate hydrogel stability and can be toxic to cell growth. Amino acid supplementation over a 20 h period via an automated feed cycle BioXpert recipe maintained nutritional levels.

#### Validation of cell viability methodology for 3-D structures

Assessing cell viability within the organoids inside alginate beads accurately was essential; individual cells could not be analysed by routine viability assays since disassociation of organoids to single cells was a destructive process breaking cell-to-cell contacts e.g. junctional complexes and desmosomes which created tissue like structures (hence organoids), and were not simply an aggregation of cells.

**Figure 2:**
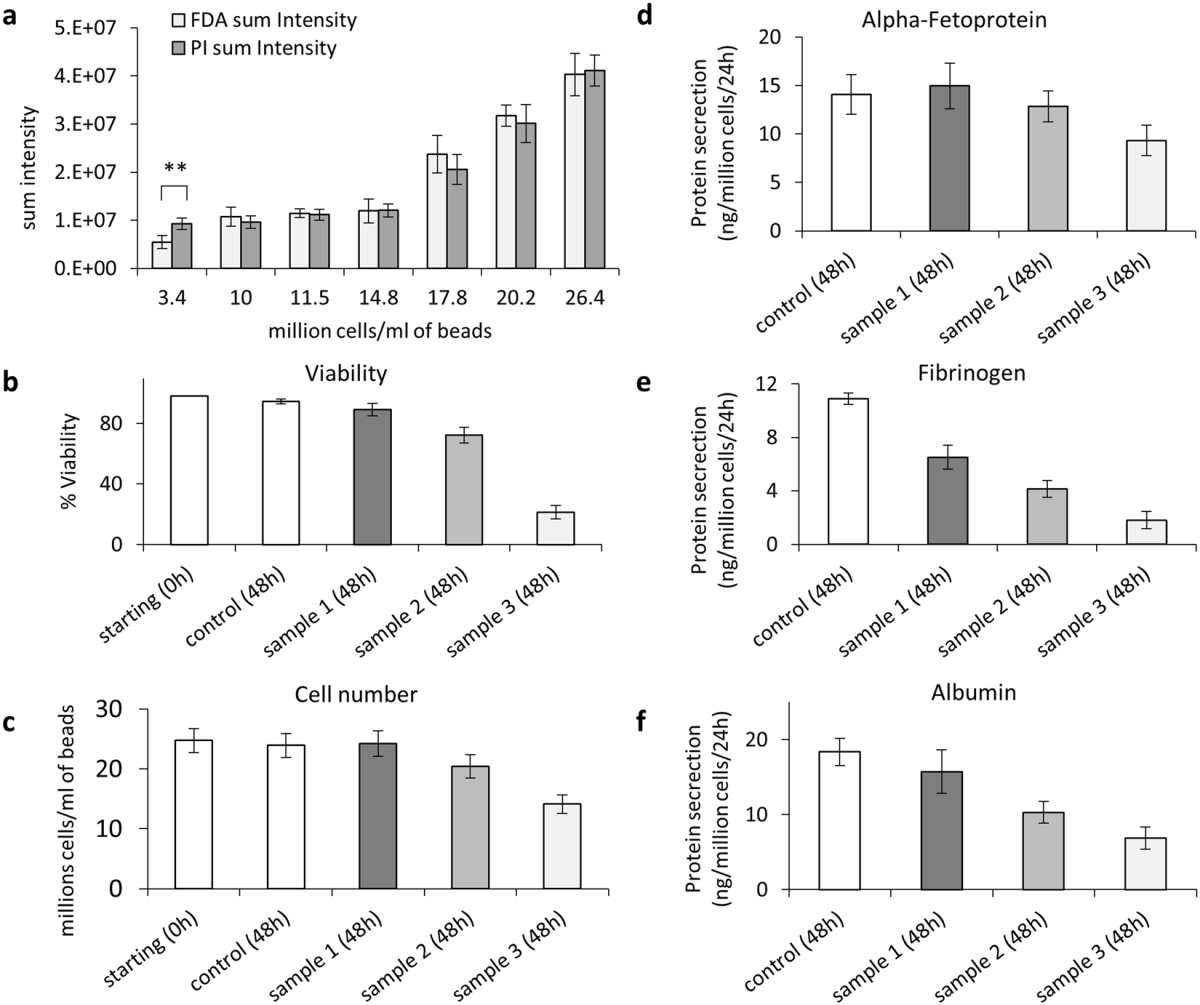
(**a**) Quantitation of fluorescent signal for cell spheroid viability assay. Sum intensity of FDA and PI signal for alginate encapsulated cell spheroids (AELS) of increasing cell density, indicating that during the whole bioprocess production the quantitation of live/dead cells was insensitive to changes in cell density, demonstrating equivalency of intensity of PI/FDA output, with the exception of the lowest cell density (difference between FDA and PI signal at 3.4 million cells per ml beads, p < 0.05). (**b**) Viability determined by image analysis compared to cell number (**c**), protein secretion (**d**–**f**) Sample 1, 2 and 3, treated with high Me_2_SO to progressively reduce cell viability (5, 10, and 15 minutes in ice-cold 50% Me_2_SO (w/v)) and a positive control (untreated cells) were tested 48 hours after treatment. Viability, cell number and protein secretion per cell decreased for samples in the order 1–3. Data was n = 5, ± SD.

To assess viable cell-number in 3D organoids we adapted the *in situ* fluorescence assay of metabolic viability, utilising FDA/PI as previously described. Quantification was made via image analysis. To ensure accuracy and robustness throughout the given cell density ranges during the growth cycle, validation with respect to the method itself, i.e. sum intensity measurements, and correlation with other assays of metabolic function are shown. Cells within the beads proliferated and remained viable during the production process, n = 21, (Fig. 1b–d). Comparison of total cell number with viability and cell function are proportional (Fig. 2).

The highest signal-to-noise ratio for FDA and PI was reached with exposure times of 150 ms and one second, respectively, with average low threshold for both FDA and PI of 41. PI exposure times of 600 ms and two seconds resulted in low signal-to-noise ratios (SNR), either being too short to generate a signal similar to that of FDA without including a high percentage of background pixels or too high, meaning that only a few cells (pixels) had to be selected. The chosen set up was confirmed with independent samples comparing AELS (same origin) of either 99% or 0% viability. There was no significant difference in sum intensity between PI and FDA for samples with cell density > 10 000 cells/ml of beads, but FDA intensity was lower in comparison to PI for samples of low cell density (3.4 million cells per ml beads, typically seen only on day 1–2 of culture) (Fig. 2a). Viability was also compared to non-invasive liver protein secretion (secretion/number cells), cell number and MTT data: value positive control > value sample 1 > value sample 2 > value sample 3, with the exception that for cell number (positive control vs. sample 1, Fig. 2c) and for MTT data (sample 1 to 3) no, or only little distinction between samples was detected (thus graphical data not shown). Alpha-fetoprotein secretion was markedly reduced in samples 1–3 up to 24 hours after treatment, but upregulated 24 hours later, while fibrinogen and albumin was further reduced or remained at a similar level (Fig. 2d–f).

Image analysis is a simple, rapid and economical tool to assess cell-bead viability without the need to disrupt either the alginate matrix or cell spheroids. Additionally, there is little scope for variability between operators - an important aspect for quality control. When taking TIF images of fluorescent signal, exposure time is recorded and an established threshold setting means that intensity is objectively quantified; this contrasts with trypan blue exclusion analysis, when a dead/viable cell count is subjective. Simultaneous staining with FDA and PI allows calculating the viability of a single bead. Typically our data was collected from at least 100 beads per condition or time-point.

#### Oxygenation of the FBB

Oxygen provision in the FBB process was from two sources. A BioXpert recipe controlled process in the SUB stirred tank reactor from 21–35% via one dissolved oxygen probe (DO1), and then from additional oxygenation direct to the biomass containing chamber. Both were controlled from post-chamber dissolved oxygen monitoring (DO2), ensuring sufficient oxygenation was afforded to biomass throughout the chamber (11.79 +/− 3.5% (mean +/− SD, n = 15), with a non-toxic concentration to the base of the chamber from the SUB. Using the oxygen flow rate, transfer across the chamber and difference between DO1 and DO2 readings, the oxygen consumption was calculated for biomass on day 12, i.e. at performance-competence, to be 0.153 ± 0.07 fmol/cell/second, n = 14. This value is greater than observed in our previous study (0.038) and from published values of other bioreactors based on different adherent scaffolds at 0.0166 fmol/cell/second)^12^ or hollow fibre cartridge (0.0028 fmol/cell/second)^13^. This is likely due to improved nutrient and oxygen controlled addition in our BAL in the GMP compliant single-use process, with better cell sampling in this design, compared with other systems, (hollow fibre and adherent scaffold processes can only achieve direct cell number sampling destructively at the end of a process, whereas the FBB enables actual bead sampling on a regular basis). We have previously shown no lack of oxygenation across the beads, with proliferation of cells equal throughout the bead and no evidence of necrotic centres on histology or electron microscopy^14^).

#### Patient safety aspects of the BAL device

The alginate encapsulated biomass was prevented from entering the patient in several ways. Firstly the biomass was contained in the fluidising chamber by 200 µm mesh at both inflow and outflow; given the size of the beads (~500 µm) intact beads could not escape the chamber. However, that may not preclude cell release, if, for example, beads were damaged. Thus two filters were sited downstream of the BAL chamber on the patient return side. The first was a depth filter that removed particulates and also bound DNA and endotoxin due to its charged nature, and the second downstream of that was a 0.65 µm filter that would prevent any possible additional cell debris from entering the patient. Important as it is that unwanted components are removed, that should not be achieved at the expense of removal of the positive components synthesised by the biomass; thus we demonstrated there was no loss of synthesised protein levels after the filter components of the BAL using fibrinogen (340 kDa), alpha 1 acid glycoprotein (41–43-kDa) or alpha 1 antitrypsin (52 kDa) as markers measured over 8 h perfusion. To ensure that the filters did not add any toxic components to the recirculating plasma, cells cultured in plasma taken before and after the filters, assayed for metabolic activity (MTT assay 8 and 24 h after exposure), indicated no differences (data not shown). Particle analysis using NanoSight technology (Malvern Ltd, Salisbury, UK) indicated removal of all particles above 0.6 µm. This meets the requirements for large volume injectables of <12/ml of >10 um and <2/ml of >25 um^15^. Finally, since the BAL extracorporeal circuit is a secondary process device not attached directly to the patient, the plasma exchange (apheresis) machine affords a final patient protection component.

#### Immunological safety aspects

**Figure 3:**
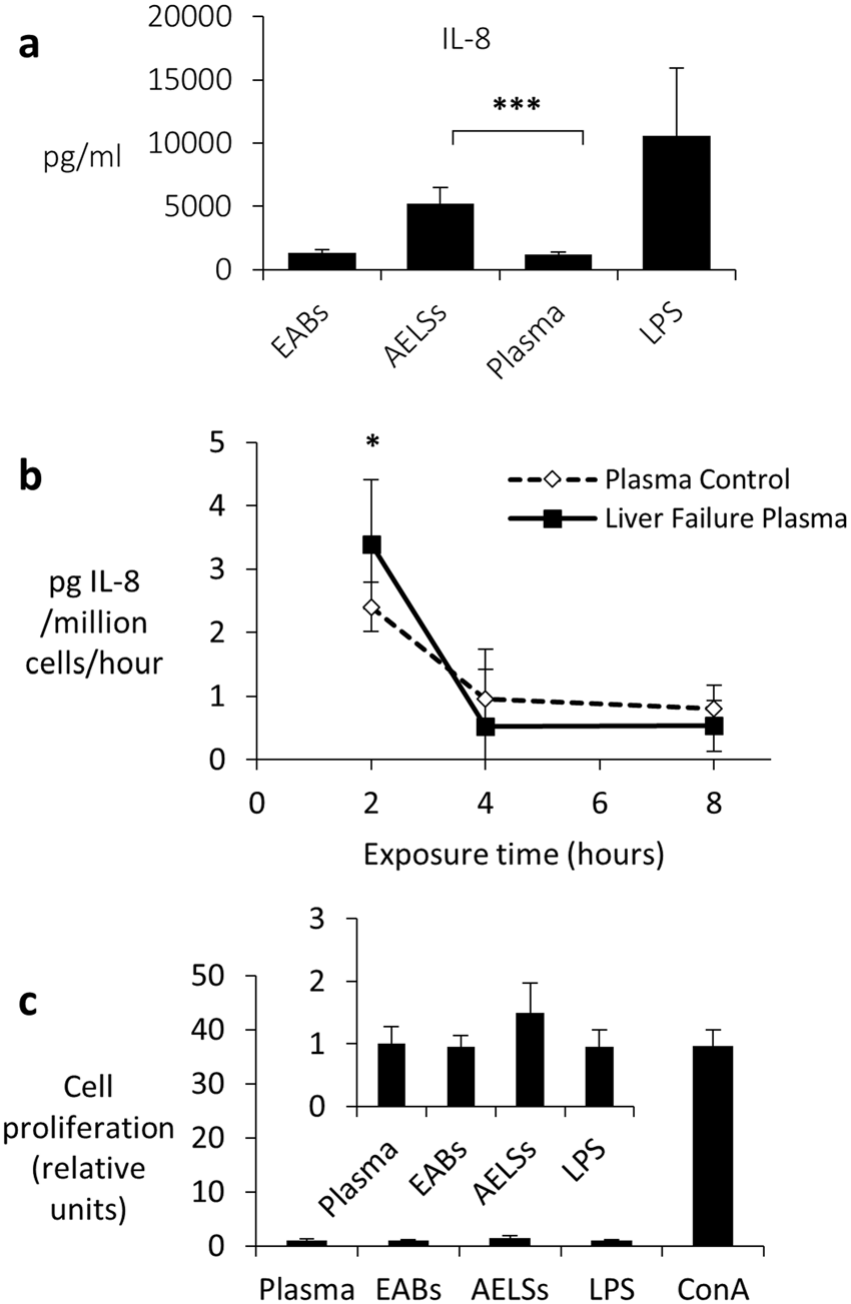
(**a**) Cytokine response of peripheral blood human leukocytes to alginate encapsulate liver cell spheroids (AELSs) and empty alginate beads (EABs). Plasma conditioned with HepG2-beads (AELS), or empty beads (EABs) was exposed for 24 h to human leukocytes using LPS as positive control. Cytokine concentrations of IL-1β, IL-6, IL-10, TNFα, IL-12p70, IL-2, IL-4, IL-5, IFN-γ, IL-17A, GM-CSF and IL-8 in the leukocyte-exposed conditioned plasma were analysed by FACS using a multiplex bead kit (BD™ Cytometric Bead Array (CBA) Human Inflammatory Cytokine Kit, Cat No. 551811; and Human Th1/Th2 Cytokine Kit, Cat. No. 550749; Human IL-17A Flex Set, Cat. No. 560383; Human IL-8 Flex Set, Cat. No. 558277; and Human GM-CSF Flex Set, Cat. No. 558335). (n = 12 in three independent experiments, ±S.D); Student’s T-test, ***P < 0.001. Only IL-8 was significantly increased in leukocytes exposed to biomass conditioned plasma. (**b)** Biomass production of IL-8 after exposure to liver failure plasma. Normal plasma obtained from healthy volunteers; liver failure plasma from two patients, (n = 4 replicates). Starting levels in LFP were higher than in normal plasma (patient 1 had 261 pg/ml and patient 2, 197 pg/ml). However, IL-8 was released from BAL biomass in both normal (2.24 pg/million cells/h) and liver failure plasma at 3.39 pg/million cells/h after 2 h of exposure at similar levels. Levels stabilised by 4 h exposure to < 1 pg/million cells/hour. Thus the environment of LFP did not impact on biomass release. (**c)** PBMC proliferation is not enhanced by empty bead or cell-bead conditioned plasma. 1 × 10^5^ PBMCs/well were incubated with normal plasma (Plasma), EAB conditioned plasma, AELSs conditioned plasma, normal plasma containing 500 ng/ml lipopolysaccharide (LPS) or RPMI medium containing 2 ug/ml Concanavalin A (ConA) for 48 h, and assessed for proliferation. Values are relative to the negative control (Plasma alone), ConA showed expected high proliferation, n = 4 replicates, ± SD, representative of three separate experiments.

Cytokine release from the biomass could potentially adversely affect the patient were it to return to the patient from the extracorporeal circuit. Cytokine concentrations of IL-1β, IL-6, IL-10, TNFα, IL-12p70, IL-2, IL-4, IL-5, IFN-γ, IL-17A and GM-CSF were undetectable in the leukocyte-exposed biomass-conditioned plasma; only IL-8 was increased compared with plasma alone or empty beads (Fig. 3a). Of the 12 pro and anti-inflammatory cytokines measured, only IL8 was detectable from **direct** biomass release (Fig. 3b), and whilst starting levels in liver failure plasma were high compared with normal plasma, the production and release over time was not different in the presence of normal or liver failure plasma. Addressing the immune response from a different perspective, proliferation of peripheral blood mononuclear cells (PBMCs), no significant increase in cell proliferation was observed when biomass was exposed compared with plasma alone or empty alginate beads (Fig. 3c).

#### Biomass viability and performance in liver failure plasma

Viability dropped by no more than 10% during 8 hours exposure to the plasma in each *in vitro* experiment, from 91.3% to 87.2% and 96.8% to 86.6% in the bilirubin-spiked plasma experiments, and from 96.3% to 92.0% after exposure to the liver failure plasma (average 6.2 ± 2%). In addition, at the end of *in vivo* BAL treatment, in pigs with liver failure, BAL biomass viability was still 93.0 ± 3.2%; n = 6, ± SD, adding weight to the lack of cell damage seen in *in vitro* exposure. The fact that the viability of the biomass was not significantly reduced over the course of BAL treatment *in vivo*, nor in presence of human liver failure plasma *in vitro*, provides good evidence that in patients it would also withstand the toxic milieu of circulating liver failure plasma. Analysis of Alkaline Phosphatase (Alk Phos), ALT and AST in the treated plasma was unaltered throughout 8 h plasma exposure, indicating lack of cell spheroid damage in presence of liver failure plasma. This is important since there are previous reports of liver failure plasma being toxic to cells in monolayer culture^16^ and also in some other BioArtificial liver experimental models^17^.

Cytochrome P450 activity was demonstrated in biomass (HepG2 3D culture in FBB) and compared with primary human hepatocytes. The specific activities were 0.071 in primary human hepatocytes with a range of 0.022–0.167 (n = 9) pmoles d-luciferase/min/mg protein. In FBB biomass there was 0.041 +/− 0.02 pmoles d-luciferase/min/mg protein. This contrasted with the same cells in monolayer culture where specific activities were 0.019. (Fig. 2 supplementary)

A functional value of bilirubin conjugation was calculated, based on the total number of cells used and volume of plasma treated. About 5 mg conjugated bilirubin is excreted in urine in adults per day (assuming 1 × 10^11^ total liver cells)^18^. In liver failure plasma, the biomass showed 88.2 mg conjugated/1 × 10^11^ cells/day.

**Figure 4:**
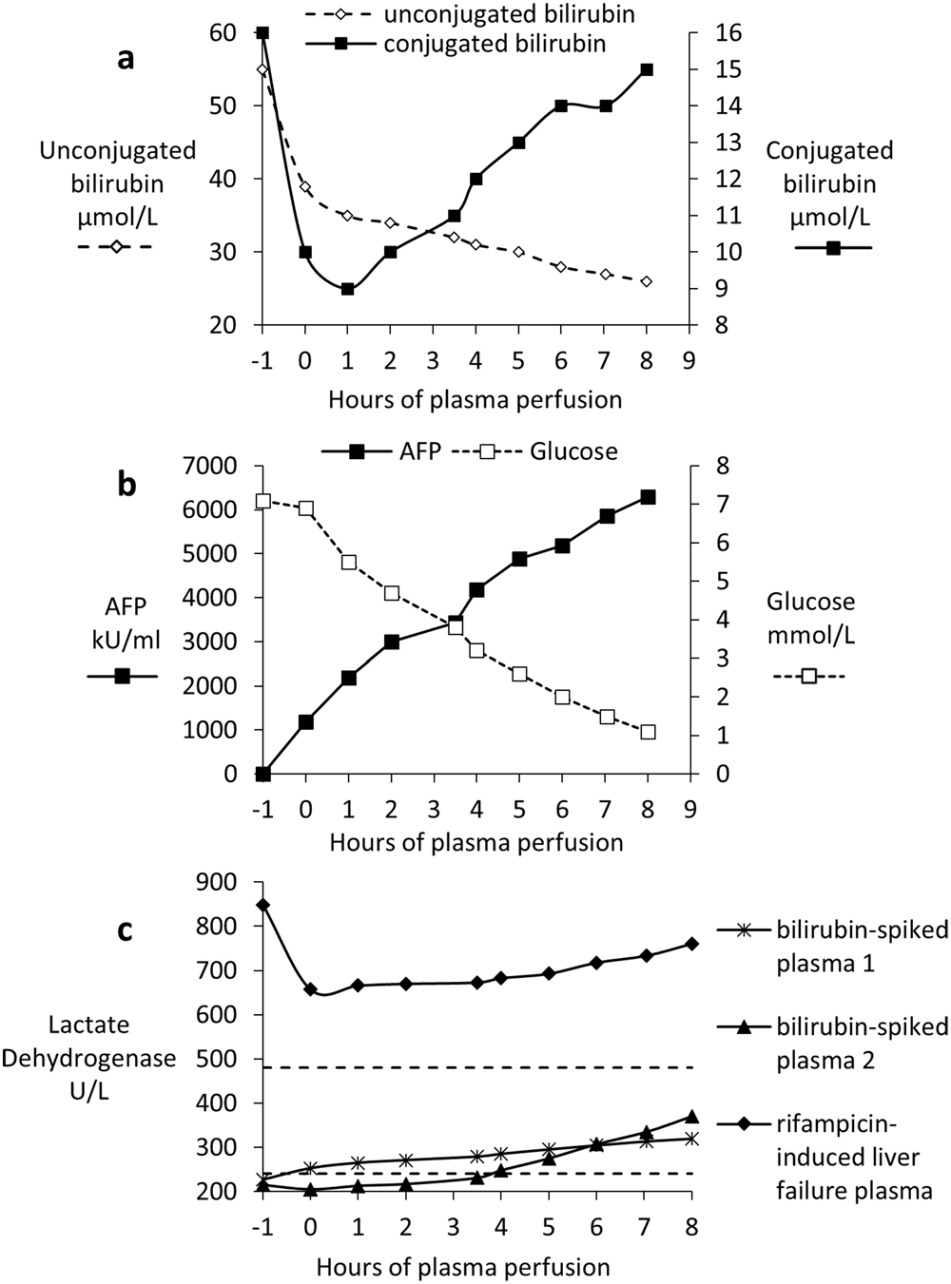
(**a**–**c**) *In vitro* testing of biomass exposed to bilirubin-spiked normal human fresh frozen plasma (FFP) or patient liver failure plasma over an 8 h period. (**a**) Biomass conjugation of bilirubin in rifampicin-induced liver failure plasma. The initial drop in both unconjugated and conjugated seen between −1 and 0 and 1 hours reflects a dilution effect of plasma with fluid within the hydrogel. From 1 h onwards, conjugated bilirubin increases and unconjugated bilirubin falls. (**b**) AFP production and glucose consumption measurements in liver failure plasma; note the linearity over the perfusion time course, an indication of continued metabolic viability and functionality. (**c**) Lactate Dehydrogenase (LD) measurements in two experiments with bilirubin-spiked fresh frozen (FFP) plasma and in liver failure plasma. Dashed lines show the normal range of LDH between 240–480 units/L.

AFP production and glucose consumption was linear during the 8 h treatment, (Fig. 4b) in normal plasma with bilirubin spike and in liver failure plasma, indicating continued function. AFP was used as a marker specific to the HepG2 cells and not present in normal human plasma, thus easily differentiating between biomass produced proteins and those found in normal human plasma. We can readily use AFP to monitor protein synthesis and release by the biomass when cultured in human plasma in patients, as a surrogate marker of synthetic function having previously demonstrated production of five liver specific secreted proteins^2^.

The bilirubin-spiked plasma experiments had additional glucose supplementation (10 mM).

A slow but non-significant rise in lactate dehydrogenase (LDH) concentration (Fig. 4c) was observed over 8 hours of incubation with bilirubin-spiked plasma, remaining well within the normal range (range shown as dashed horizontal line). As expected, the patient with acute liver failure had very high LDH levels initially, indicating liver cell damage in the patient; however, rate of release during the 8 h treatment was not different to normal plasma, indicating that the liver failure plasma was not damaging the biomass.

As lactate dehydrogenase is a measure of cell damage we could not have expected to “correct” the values in the liver failure plasma as the dead/dying cells would have been in the patient at the time the plasma was collected. These results provide additional evidence that the functional capacity of the biomass was not compromised by liver failure plasma.

#### Porcine ischaemic acute liver failure treated with the BioArtificial liver in vivo

The short term cold/ambient chain storage solution allowed the biomass to be delivered to the site of the animal trial > 9000 km (6000 miles) from site of production.

All animals in both groups had comparable biochemistry and haemodynamic values prior to connection of the BAL circuit (supplementary data Tables 1 and 2), with the exception of AST which was higher in the group allocated to cell treatment (AST control group = 150 ± 26, n = 16; AST, cell treated group = 311 ± 47, n = 13); whilst this is unusual, that it is in the cell-treated group obviates against any bias suggesting improvement in that Group is due to them having undergone less liver damage; all 17 other parameters did not differ between the groups at this point. Ischaemic surgery was performed blind with respect to BAL treatment.

**Figure 5:**
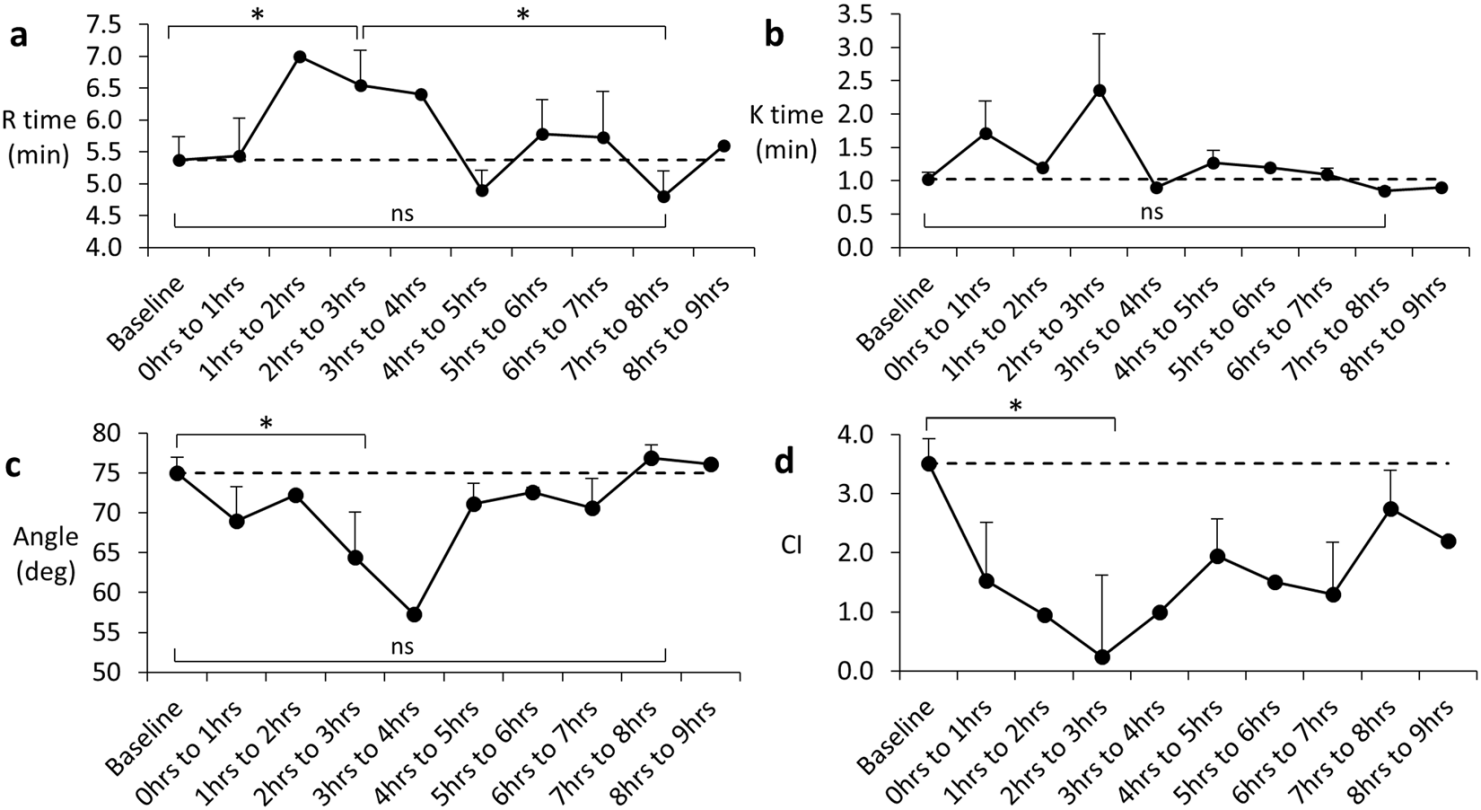
(**a**–**d**) Assessment of clotting function in Cell-BAL treated pigs after induction of liver failure, before and after BAL addition. Averaged R time, K time, Angle and Coagulation Index (CI) of Cell-BAL treated pigs (± SEM, n = 13); where no error bars are shown, that data point does not have replicates, due to occasional technical issues with blood sample analyses. Dashed line indicates baseline normal value. Student’s t-test, *p < 0.05.

Coagulation was assessed by two different methodologies. Prothrombin time and related INR measured by routine chemical pathology (Pathcare Pty, South Africa), indicated a significant improvement in the Cell-BAL treated group. The control group prothrombin times were 106.1 ± 14 sec (n = 10, ± SEM) and INR of 7.6 ± 1 (n = 9, ± SEM) at 6–7 h from ischaemia, but these were reduced to 52.2 ± 11 sec (n = 10, ± SEM) and 4.4 ± 1 (n = 9, ± SEM) in the cell-treated group (and Fig. 3 supplementary). This improvement in coagulation was confirmed using Thromboelastography, a serial test of several kinetic parameters associated with blood clotting (Fig. 5). The data illustrates initially a worsening in clot kinetics, (e.g. increased R time - time to clot initiation, slower time taken to achieve a certain clot strength after initiation - k time and angle), and coagulation index, CI). These were followed by improvement once attached to the Cell-BAL back towards normal. CI reflects all of these parameters together in a calculated indicator of overall coagulation^19^ as shown in Fig. 5d. The dramatic improvement of clotting parameters e.g. prothrombin time and INR in Cell-BAL treated group, but not the control group, provides good evidence that the normal plasma used to prime the BAL system was not responsible for the efficacy since both test and control group were identical except that the control group contained empty alginate beads (no cells). Moreover, human proteins including fibrinogen and prothrombin were demonstrable in the pig plasma. Since the liver was removed from the circulation, but left *in situ*, the only source of improved function could be the biomass.

**Figure 6:**
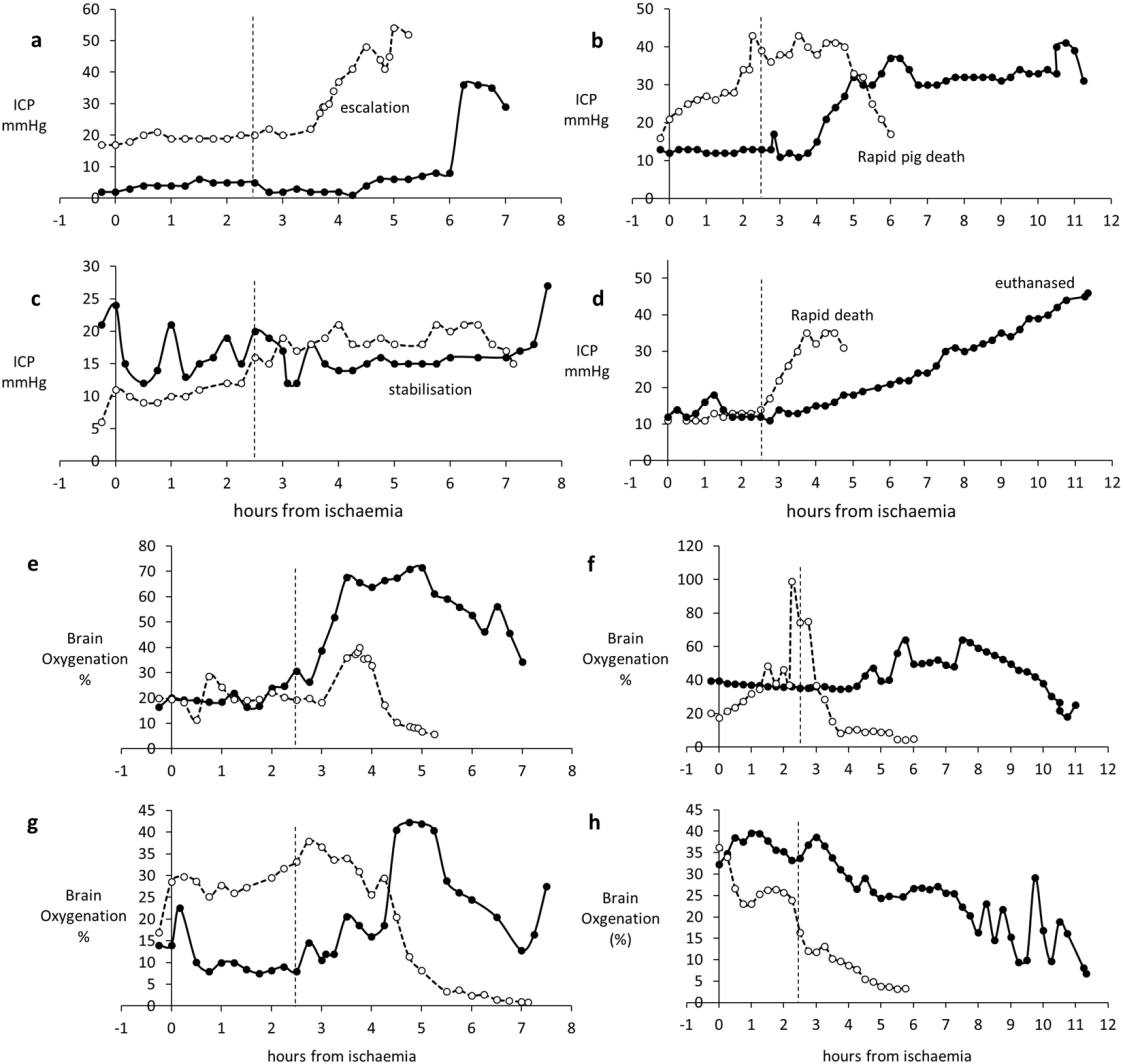
Parameters of brain function in four pairs of control and Cell-BAL: animals matched for severity of ammonia levels before time of attachment to BAL, as ammonia is a critical precipitant of brain dysfunction. BAL addition: vertical dashed line. Control-BAL - ○, dashed; Cell-BAL - ●, solid**:** (a–d) Intracranial pressure (ICP) Examples show the ICP stabilisation enabled by Cell-BAL, (**e–h)** Brain oxygenation in control and Cell-BAL: Examples show the increased brain oxygenation enabled by Cell-BAL.

A rise in intracranial pressure (ICP), often leading to coma and death in patients, is an important prognostic indicator of a fatal outcome in acute liver failure (ALF) in man. There was a variable response between pigs, as also occurs in patients, Cell-BAL treated pigs showed either a reduction or stabilisation of ICP that was confirmed by appropriate concomitant changes in brain oxygenation measurements; these two parameters were measured using independent probes and monitors, providing robust evidence that both parameters were indeed improved (Fig. 6). As a rapidly rising intracranial pressure and associated cerebral oedema leads to death, the ability to stabilise and even decrease the intracranial pressure has significant potential clinical benefits in stabilising patients with ALF who are awaiting a donor. A rapidly rising intracranial pressure and associated cerebral oedema usually precludes liver transplantation.

**Figure 7:**
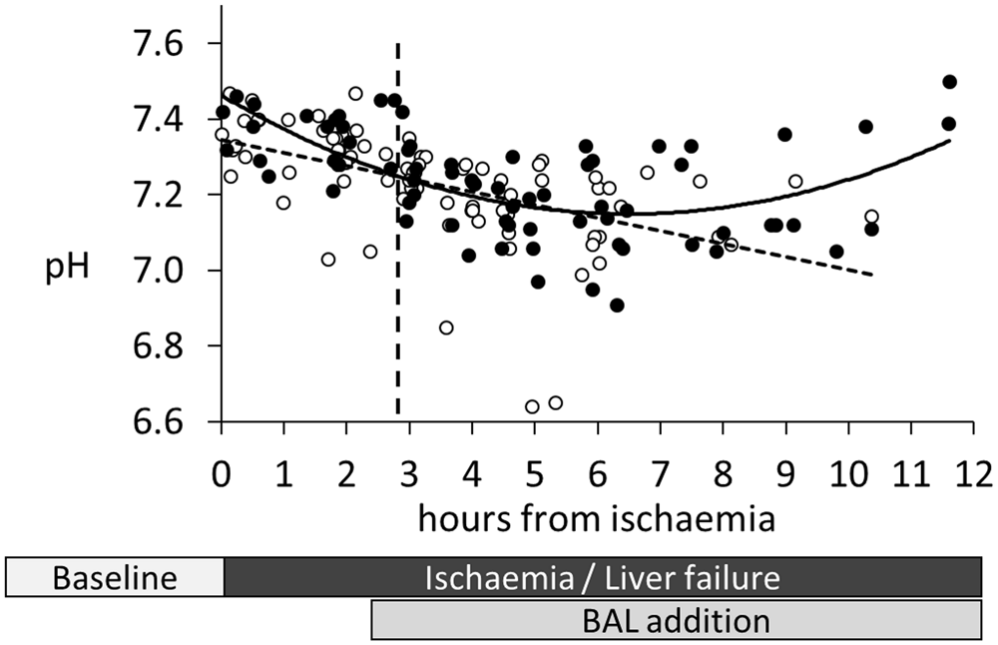
Acidosis in pigs: pH measurement. Control-BAL - ○, dashed trendline, n = 15; cell-BAL - ●, solid trend line, n = 13. BAL addition – vertical dashed line. 0.5 ml whole arterial blood was collected into heparinised syringes, and 0.2 ml analysed for blood gases including pH using a Radiometer automated analyser.

Metabolic homeostasis is disturbed in liver failure, with a fall in pH and bicarbonate and a rise in anion gap and lactate, leading to acidosis, the severity of which is a prognostic sign in ALF in man. Figure 7 illustrates the changes in pH over the course of liver failure induction and treatment. In the control group pH continued to worsen, whilst in the Cell-BAL treated group pH improved back towards normal.

The need for vasopressor treatment arises in patients with haemodynamic instability; the higher the dose required the less able are patients to withstand the rigours of major surgery, such as organ transplant.

**Figure 8:**
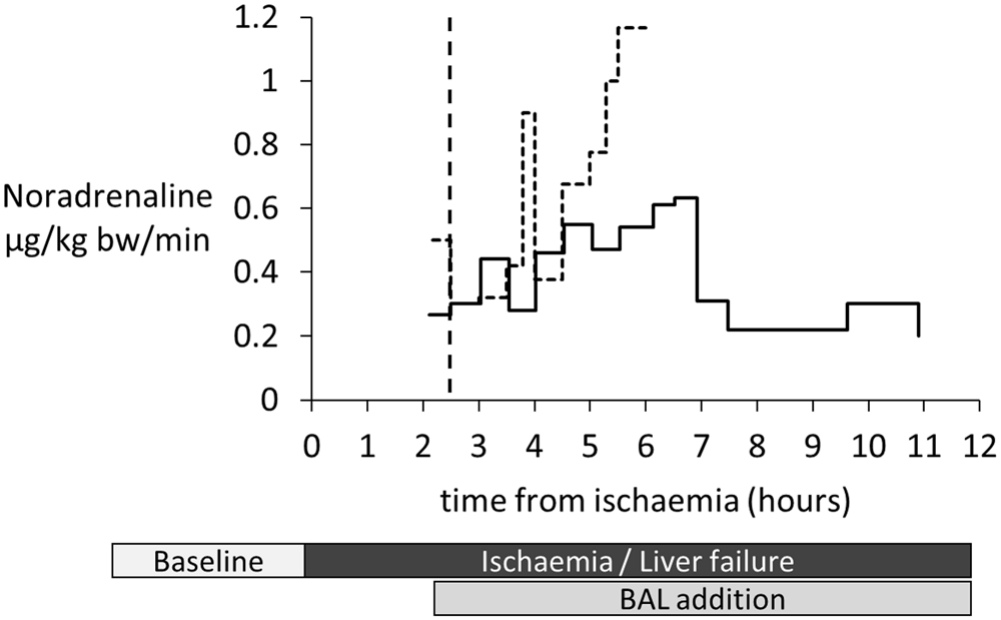
Vasopressor requirement: Both Cell-BAL treated (solid line, n = 8) and Control-BAL treated (dashed line, n = 6) pigs received noradrenaline infusions into the external jugular vein from 30 min before BAL addition. Dose escalation maintained a cerebral perfusion pressure of 60 mmHg.

Figure 8 shows the comparative requirements for the two groups of pigs requiring noradrenaline to maintain mean systolic pressure, indicating a dramatic drop in requirements in the Cell-BAL treated group, compared to those in the empty-bead BAL treated group. Since supportive inotropes were used, similar haemodynamic stability was recorded in both groups, however the biologically important difference is the lesser requirement for inotropic support in the cell BAL-treated group.

All but one animal in each group became anuric (<20 ml/h urine output). All animals developed pulmonary oedema evidenced by increased extravascular lung water (Picco ELWI normal range 3–7mls/kg; average in control group over period of ischaemia was 12+/− 2 mls/kg (EVLW 365+/−49) at time of BAL addition and 12+/−3 at last time point (EVLW 366+/−102) and similarly (as expected in a permanent liver failure model) ELWI was 13+/−3 (EVLW 395+/−88) and ELWI 12+/−3 (EVLW 365+/−81) at time of Cell-BAL addition and last thermodilution time point respectively.

Furthermore, increasing levels of positive pressure ventilation were required providing further pulmonary oedema. Extravascular lung water index is derived from thermo-dilution studies in the Picco performed intermittently, and only possible after the arterial catheterization which occurred during the establishment of liver failure surgical model.

This model of acute liver failure in the pigs has strong parallels to ALF in man. It was complicated by multi-organ failure as manifested by myocardial failure requiring increasing inotropic support, renal impairment with increasing oliguria and eventual anuria leading to fluid overload with pulmonary oedema. Whilst it was not possible to assess for peripheral oedema in the skin of pigs, they did develop conjunctival oedema. At the time of demise, animals had developed significant ascites. Clinically multi-organ failure is often the cause of death in patients, who become increasingly oliguric, eventually anuric and often require renal support usually in the form of continuous veno-venous haemodialysis to avoid significant fluid shifts that can exacerbate raised intracranial pressure. They also develop adult respiratory distress syndrome and myocardial failure. Other patients develop life threatening raised intracranial pressure and cerebral oedema.

**Figure 9:**
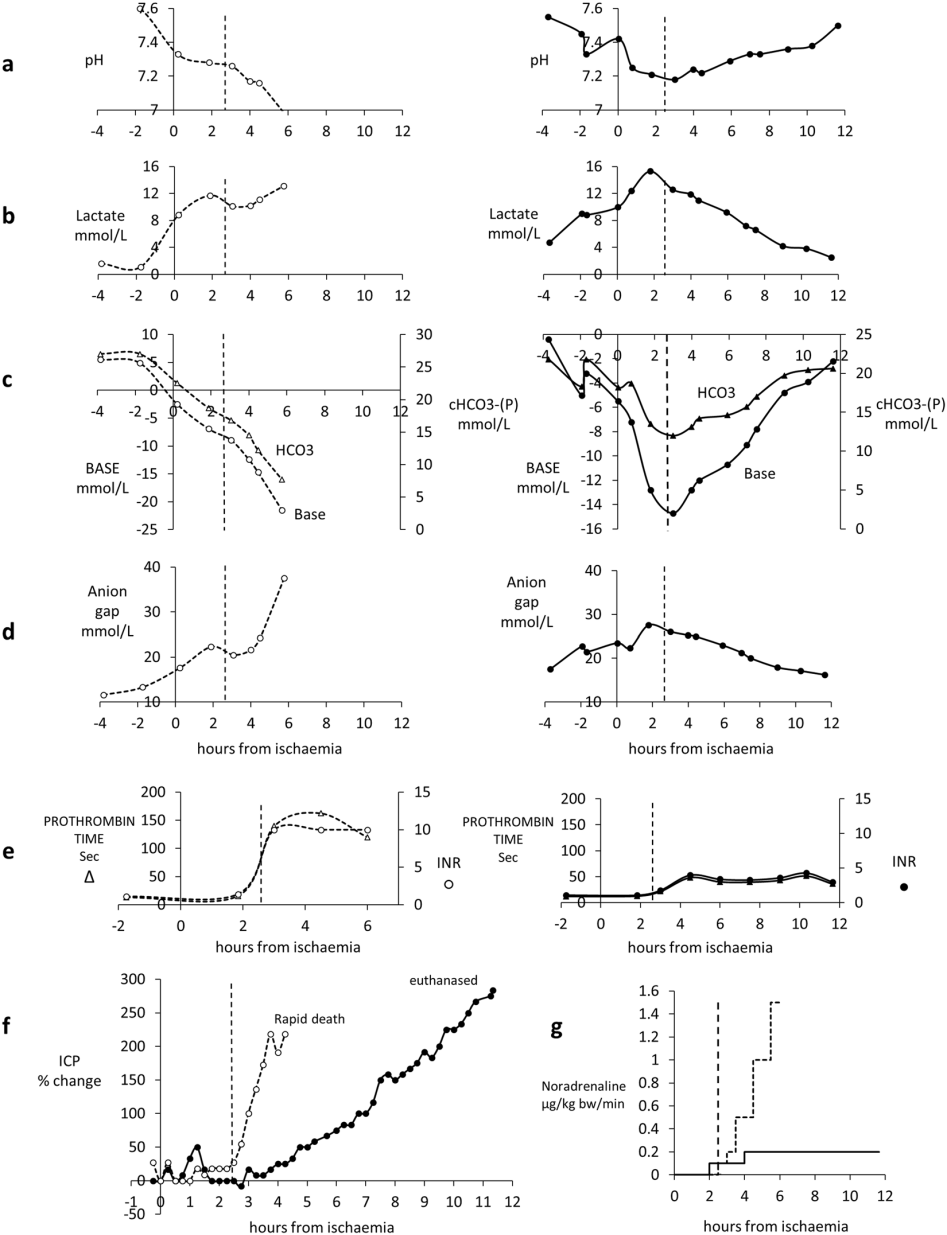
(**a**–**g**) Comparison of parameters of severity of acute liver failure in control empty bead BAL (-o-) treated pig vs. Cell-BAL treated pig (•), with similar degrees of liver dysfunction prior to connection to the BAL. BAL treatment started at 2.5 h. Both pigs had same ammonia levels at connection to the BAL. Control-treated pig survived 6 h after liver failure induction, Cell-treated pig survived >12 h after liver failure induction. Vertical dashed line signifies time at which BAL was added. (**a**) pH (**b**) Lactate. (**c**) Base and bicarbonate. (**d**) Anion gap. (**e**) Prothrombin time and INR. (**f**) ICP change from baseline. Control-BAL (○) exhibited a rapid rise in ICP compared with Cell-BAL (●), i.e. Control-BAL 154% change from baseline per hour; Cell-BAL 25% change from baseline per hour. (**g**) Vasopressor requirements: noradrenaline was administered IV at rates required targeting a cerebral perfusion pressure (CPP) of 60 mmHg, so the blood pressure was optimised depending on the ICP.

We show in Fig. 9, an example of a single pig pair matched for ammonia at the time of addition of BAL. In each case, the control as expected showed worsening of parameters of acidosis, whilst with Cell-BAL treatment the parameters all improved after BAL addition.

**Figure 10:**
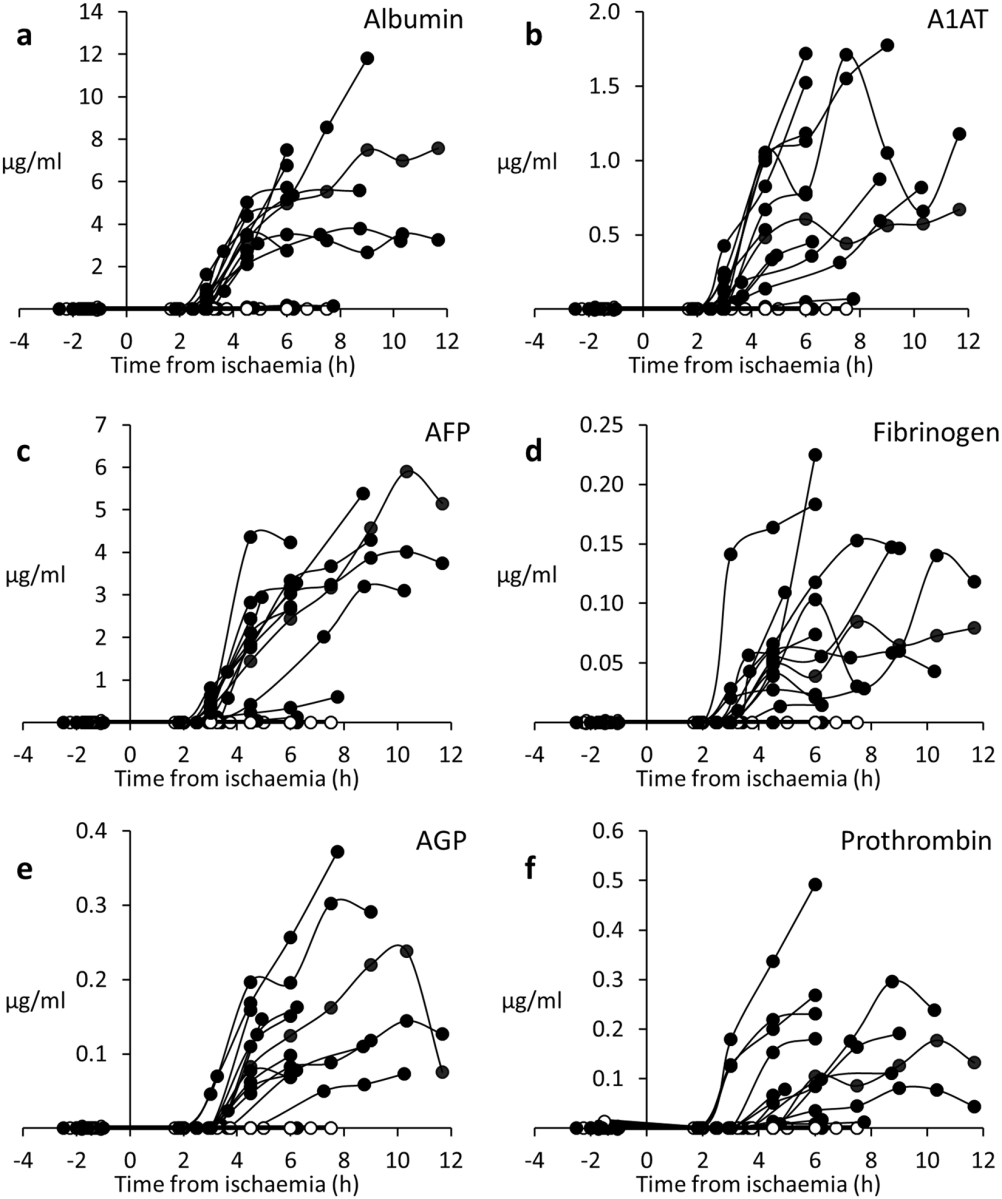
(**a–e**) Human proteins detected in pig plasma *in vivo*. Human proteins were produced by cell-BALs (-●-) during treatment of ischaemia-induced liver failure pigs, but not in control-BAL treated (-○-) animals; the graphs show concentrations that reflect BAL biomass production and any utilisation of those proteins by the pig; data are shown as concentrations in whole system, taken from pig blood samples and AUC analysis is also shown in legend. Proteins assessed included (**a**) Albumin (AUC: 136.7 ± 35 total mg produced over treatment by cell-BALs;), (**b**) Alpha-1-Antitrypsin (A1AT; AUC 24.1 ± 5 mg), (**c**) Alpha-Fetoprotein (AFP; AUC 90.9 ± 22 mg), (**d**) Fibrinogen (AUC 2.6 ± 1 mg), (**e**) Alpha-1-Acid Glycoprotein (AGP; AUC 4.2 ± 1 mg), (**f**) Prothrombin (Pro: AUC 4.2 ± 1 mg); n = 13, ± SD. Human proteins in Control-BAL treated pigs (-○-) were undetectable.

Human proteins were detectable in pig blood samples in the Cell-BAL treated, but not Control BAL (Empty bead)-treated pigs (Fig. 10), after BAL addition. Five liver specific secreted proteins were assessed, as well as alpha-fetoprotein, a protein not seen in normal blood plasma, but specifically produced by the biomass. AFP will be a useful marker of continued biomass function in human trials as it can be assessed by routine chemical pathology, whilst biomass production of other human plasma proteins, present in the plasma, is difficult to quantify in the setting of high concentrations of these proteins already and normally present in the patient plasma. The Rotem TEG results (Supplementary Figure 4) confirm synthesis of fibrinogen.

## Conclusions

The UCLBAL was efficacious in a porcine model of ischaemic acute liver failure using pigs of adequate size to reflect the same liver size as expected in man (25.2–34.4 kg pig weights, 0.88–1.71 kg liver weights, n = 25). This is indicative of a good chance of success in man. The model chosen was deliberately extremely severe, and irreversible, thus removing the possibility of survival as an endpoint. The development of multi-organ failure is the common terminal event in patients presenting with acute liver failure and the development of MOF precludes liver transplantation. This confirms the severity of the acute liver failure model used and provides the opportunity to assess the potential beneficial effect of the BAL on complications that are invariably terminal and preclude transplantation. Moreover, the advantage of the model was that every animal developed severe acute liver failure, unlike some chemically induced models where a proportion of animals do not succumb to liver failure^20,21^ as demonstrated in a recent study from Shi *et al*. using D-galactosamine treated with HiHeps (derived from iPSCs)^22^.

In our surgical ischaemic model significant liver damage was evident 2 hours after ischaemia was induced, so the BAL was attached at ~2.5 h in both control and treated group (2.8 ± 0.5 h from ischaemia, n = 29). The degree of severity resulted in survival times of 5–12 h after ischaemia (Control-BAL 7.04 ± 1.9 h, n = 15 (Cell-BAL treated 8.21 ± 2.3 h, n = 13; in one control pig rendered ischaemic but not treated with BAL, survival time was 7 h; Kaplan Meier survival curve shown in Fig. 5 supplementary), such that the window of opportunity for treatment varied from 4–8 h. In man with liver failure it would be appropriate to treat for longer periods, until function of the native liver was restored, or in other patients until a donor organ becomes available (the bridge to transplant). Consequently, *in vitro* experiments exposing performance-competent cell beads to liver failure plasma for up to 167 hours are ongoing and early indications are encouraging at least up to 24 h. This will be significant in the delivery of the treatment in man, increasing the length of potential treatment with a single biocartridge. We have previously demonstrated maintained functionality in human liver failure plasma^4,23^ and the data presented in this study confirm those findings. The improvements seen were not identical in all animals, reflecting the fact that the main manifestation of ALF differed in different animals. A similar variability occurs in man, thus for example, the degree of coagulopathy was variably raised, and in some animals the abnormal parameter was predominantly cerebral. Thus, there is no single index of severity to compare between groups; nonetheless it was only in the Cell-BAL treated group that improvements were seen.

Particularly striking was the improvement in clotting time, evidenced by two sets of tests (PT/INR, and Thromboelastography) in clinical use. Notably, in the control pigs, the PT/INR rose to exceed those levels indicating an urgent need for a transplant in humans (PT > 100 secs; INR > 6), but did not in the animals treated with Cell-BAL. These results were assessed by a routine chemical pathology service (Pathcare Pty), similar to that used in any hospital setting, and validated by entirely different tests for clotting, assessing four independent parameters of clot kinetics, all demonstrating improvements.

Patients with acute liver failure often require high doses of vasopressors to maintain haemodynamic stability; this was mimicked in this porcine model, however whilst the control group required escalating does of noradrenaline, the cell-treated pigs, once established on the BAL, had a reduction or stabilisation of vasopressor requirements. In man this would translate to a significant clinical impact given that 75% of patients die from progressive multiple organ failure and require increasing inotropic support. Since there is an upper limit of vasopressor requirements above which a patient is considered too unstable for transplantation, a device that could prevent this futile pharmacological intervention would be considered highly clinically effective. Intracranial pressures were also improved and/or stabilised in pig pairs matched for ammonia levels at ischaemia (i.e. before BAL addition), and those improvements were mirrored by higher brain oxygenation, indicating better brain function, another key clinical parameter in acute liver failure, since raised ICP and associated decreased brain oxygenation and cerebral oedema are common precipitants of death. These results in pigs with human sized livers confirm and extend our previous observations^8^, using a smaller cell mass in smaller pigs which had smaller livers. In the current study, the severity of acute liver failure evidenced by prognostic clinical parameters was indeed more severe and had a more rapid progression to death, nonetheless clear evidence of effectiveness of the UCLBAL was established.

There are significant differences between the system we have developed and other aspirant technologies. Two issues appear paramount; one the cell type providing the biomass, and two, the delivery of function with a wide range of bioreactor designs being explored.

With respect to cell type, cells from different species, predominantly human or pig, have been utilised, and importantly both primary cells and proliferating cell lines have been employed.

The choice of cells for any BAL is important, balancing availability and practicality with performance and risk. Primary pig cells are readily available, however, they have both biological incompatibilities with man and biological risks. Pig-human incompatibilities may imperil protein-protein interactions. Further, in a study using porcine hepatocytes in a fluidised bed bioreactor in pigs with liver failure, whilst survival time in a D-galactosamine model was increased, there were marked perturbations in the serum metabolome, e.g. reductions in some phospholipids in particular phosphatidyl and lysophosphatidyl cholines as well as fatty acids^24^, molecules intimately associated with the stress response. It is also known that primary porcine hepatocytes are prone to loss of function and apoptosis *in vitro* ^25^.

The failure of a controlled-trial using pig cells in acute liver failure in man^26^ may well reflect these cell issues. Moreover, the use of porcine cells has the risk of transmitting porcine endogenous retroviruses (PERV), and whilst to date no patients receiving treatment with porcine cells in BioArtificial liver devices have been infected, it is clear from *in vitro* studies that it is indeed possible^27^. Gene editing has demonstrated experimentally that 62 copies of PERV pol can be deleted from PK15 cells^28^; whether that will be true of all pig cells is yet to be demonstrated.

In respect of human cells a lack of availability of primary cells renders this approach non feasible for routine clinical practice even though pilot experiments suggest it might be successful. A variety of cell lines have therefore been used. We used HepG2 cells and have evidence that in 3-D culture, the functional activity of this cell line is equal to primary hepatocytes *in situ* in many synthetic functions and even drug metabolising functions are upregulated compared with the poor performance seen in 2D culture, albeit not every function. As we have previously shown HepG2 cells do not eliminate ammonia. The urea cycle cannot be completed due lack of arginase 1 and Ornithine carbamoyl transferase^29,30^. HepG2 cells exhibit the Warburg effect^31^ of anaerobic glycolysis. Alternative proliferating cells include HepaRG cells^32,33^; these cells, similarly to HepG2 cells, significantly upregulate their performance in 3D culture^34^, however, there is controversy as to their robustness on culture in plasma. There is some evidence HepaRG cells are damaged by liver failure plasma and to a lesser extent also by normal human plasma, thus making them less appropriate for patients with acute liver failure^17^. This contrasts with our experience with HepG2 cells in the UCLBAL bioreactor which function well in normal plasma and maintain function and viability in liver failure plasma as we have shown^4^, (Coward, *et al*.^35^, Selden *et al*.^8^ and this study). Bhattacharya *et al*.^36^ have shown that the Warburg effect has been associated with drug resistance leading to a survival advantage for cells exhibiting such action. This could be an advantage for a biomass aimed at treating acute liver failure, and may go some way to explain why viability remained high in in the biomass throughout the treatment with liver failure plasma, both human, in *in vitro* experiments, and *in vivo* in the ALF model in pigs.

There are some minor functional differences between HepG2/C3A and HepaRG cells, but the only major differences are increased testosterone metabolism and contact inhibition of HepaRG cells. Theoretically, the latter might reflect a lower risk should intact cells reach the patient circulation. However, other strategies such as filters to exclude cells, particles and DNA re-entering the patient circulation, as are incorporated in our BAL, can obviate this risk.

One other consideration should be taken into account; the origin of the cell lines, HepG2 having been isolated from a hepatoblastoma of an adolescent male^37^, whilst HepaRG cells originate from a patient with Hepatitis C^38^.

Although theoretically human ESC or IPSC derived cells offer potential for generating cells to fill a BAL, currently the time and cost of reagents involved in providing cells at the required scale renders this approach prohibitive. Furthermore the activity of these cells still does not match that of primary cells^39^, and reflects an immature phenotype.

In respect of bioreactor design, these fall into three broad categories: the hollow fibre-based systems in which there is a diffusion barrier placed between the cells and perfusing blood or plasma, and in contrast, flow past systems where cells are immobilised on a solid matrix eg AMC BAL, and flow through systems such as fluidised bed bioreactors. These latter two have no significant diffusion barrier between plasma and cells.

ELAD (Vital Therapies) is a hollow fibre system, where only a < 100,000 Dalton fraction of the plasma is treated, and has a diffusion barrier thus reducing effective mass transfer, and a practical complication of pore blockage^40–43^. Moreover, it is impossible to sample cell function directly, rendering quality control difficult, and the hollow fibre cartridge is not cryopreservable. Flow past and flow through systems avoid the presence of a diffusion barrier. Examples of flow past systems include the AMC bioreactor which uses cells attached to a solid surface and has been trialled in man (when charged using pig hepatocytes)^44^, and more recently using HepaRG cells experimentally, but this is still limited by lack of ability to directly sample cell function for quality control and lack of cryopreservability. An alternative flow past system, with primary porcine hepatocytes immobilised on the external surface of collagen beads, was unsuccessful in human controlled clinical trial^45^.

Flow through systems, involving either single cells or cell spheroids in a fluidised bed bioreactor potentially offer the greatest scope for mass transfer between plasma and biomass^7,46,47^.

We have previously shown that the alginate encapsulation *and cell proliferation* enabling generation of 3D cell spheroids markedly enhances cell function using an alginate hydrogel without a molecular diffusion barrier such as poly-l-lysine. The period of culture to performance-competence allows the generation of endogenous and extracellular matrix proteins which contribute to the extracellular milieu of each cell.

A recent paper from Legallais *et al*.^48^ has indicated that spheroids made by physical means and then encapsulated in alginate and used in a similar fluidised bed system retain integrity, do not disturb haemodynamic stability in normal sheep, and reduce time taken for production. However protein production in their study, e.g. albumin, is approximately 200-fold lower that obtained in our UCLBAL system in plasma of pigs with liver failure (Fig. 10 legend, derived from AUC data), and in culture medium production per cell is one tenth (Legallais *et al*.^49^ <70 ng/million cells/h compared with UCL BAL >800 ng/10^6^ cells/h;). Other designs for fluidised bed bioreactors have been tried with spheroids of primary porcine hepatocytes^26^.

Our BAL design has manufacturing versatility; alginate beads containing cell spheroids could be generated to performance competence in individual chambers to be used in one patient or could be processed in bulk to charge many BALs. Cell function can be readily sampled on aliquots of alginate beads, enormously enhancing potential for quality control required of a clinical cell therapy. Cryopreservation ability offers a pathway to store and distribute the biomass as and when required for patient use^50–56^. The financial viability of a BAL as a clinical tool, of course remains to be fully established; issues such as costs savings with bulk production, as well as both clinical and cost advantages compared to the alternative therapy of liver transplantation are highly relevant considerations. Preliminary costs of goods analysis using research scale costings indicate the feasibility of the UCLBAL. Further, this alginate encapsulated cell spheroid/fluidised bed bioreactor system also offers a generic platform for three dimensional cell culture that could accommodate any proliferating epithelial cell type including cells derived from stem cells, and may have many uses in the era of cell therapy.

Most strikingly of all, the studies reported here have demonstrated efficacy in improving prognostically significant parameters of liver failure in a large animal model of severe acute liver failure using a system designed at clinical scale.

Applied in the human setting this BioArtificial Liver machine could aid the treatment of patients with acute liver failure, allowing time for regeneration of patients’ own liver, obviating the need for transplant. In patients with chronic liver failure, suffering periods of decompensation due to bleeding or sepsis it could allow time for treatment to reverse the precipitating cause and restore the status quo ante. Patients admitted to intensive care with acute decompensation are at risk of demise in >50% of cases^57^. Furthermore it could be used peri-transplantation (primary non function or delayed graft function where a newly transplanted organ fails to function immediately), or in the setting of liver surgery for metastases requiring resection greater than is usually compatible with survival, as well as resection of Hepatocellular carcinoma. In all these circumstances the BAL would provide temporary liver support.

Finally, the current trend to use extracorporeal circuits for plasma exchange to stabilise patients awaiting transplant indicates that this technology is likely to be readily acceptable for clinical uptake^58–60^, with the BAL providing a bolt-on of biological function to an extracorporeal treatment already in use.

## Electronic supplementary material

Supplementary information
