# Supplementary material for: A clinical-scale BioArtificial Liver, developed for GMP, improved clinical parameters of liver function in porcine liver failure
Source: Sci Rep. 2017 Nov 6;7:14518. doi: 10.1038/s41598-017-15021-4 (PMC5674071; doi:10.1038/s41598-017-15021-4)
Supplement: Supplementary file 1 — Supplementary information [file 41598_2017_15021_MOESM1_ESM.pdf]

Supplementary Info Selden *et al.*

A clinical-scale BioArtificial Liver developed for GMP improved clinical parameters of liver function in porcine liver failure

Clare Selden*[#], James Bundy[#], Eloy Erro[#], Eva Puschmann[#], Malcolm Miller[+], Delawir Kahn[+], Humphrey Hodgson[#], Barry Fuller^, Jordi Gonzalez-Molina[#], Aurelie Le Lay[#], Stephanie Gibbons[#], Sherri Chalmers[#], Sunil Modi[#], Amy Thomas[#], Peter Kilbride, Agnes Isaacs[+], Richard Ginsburg[+], Helen Ilsley[+], David Thomson[+], Galya Chinnery[+], Ncedile Mankahla[+], Lizel Loo[+], C.Wendy Spearman[+].

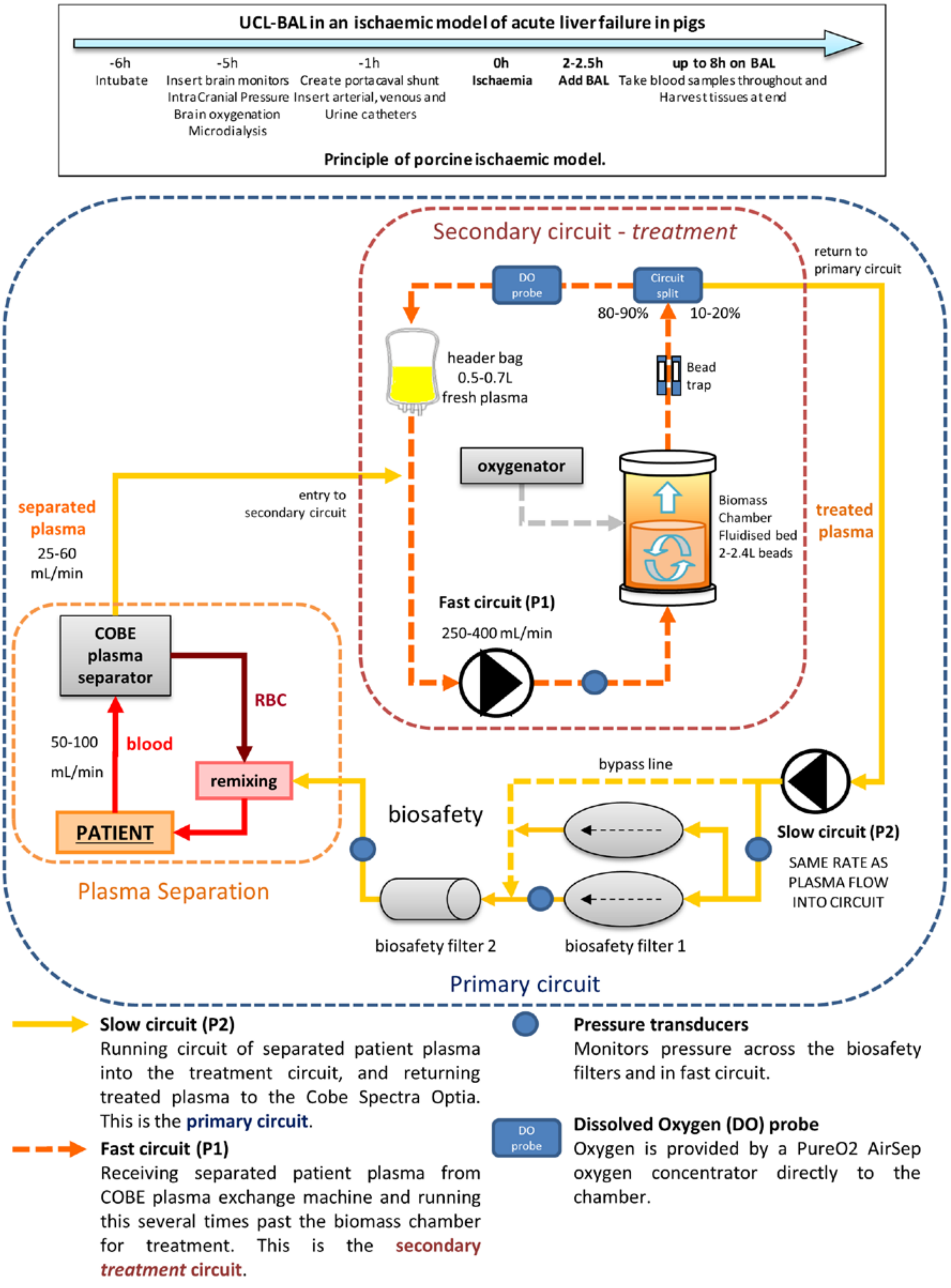

Figure 1 (Supplementary)*: Time course of pig liver failure model used for BioArtificial liver testing, and detailed circuit diagram for patient system.*

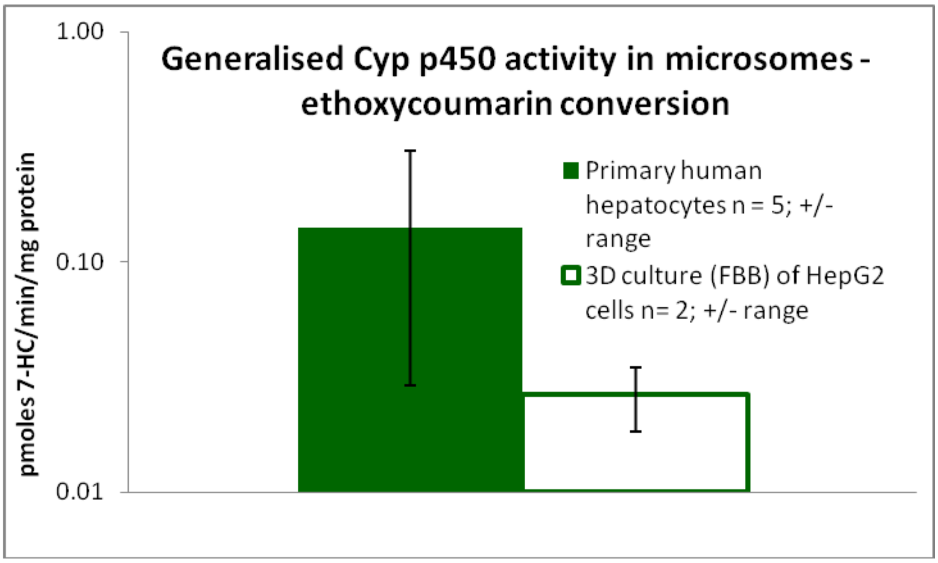

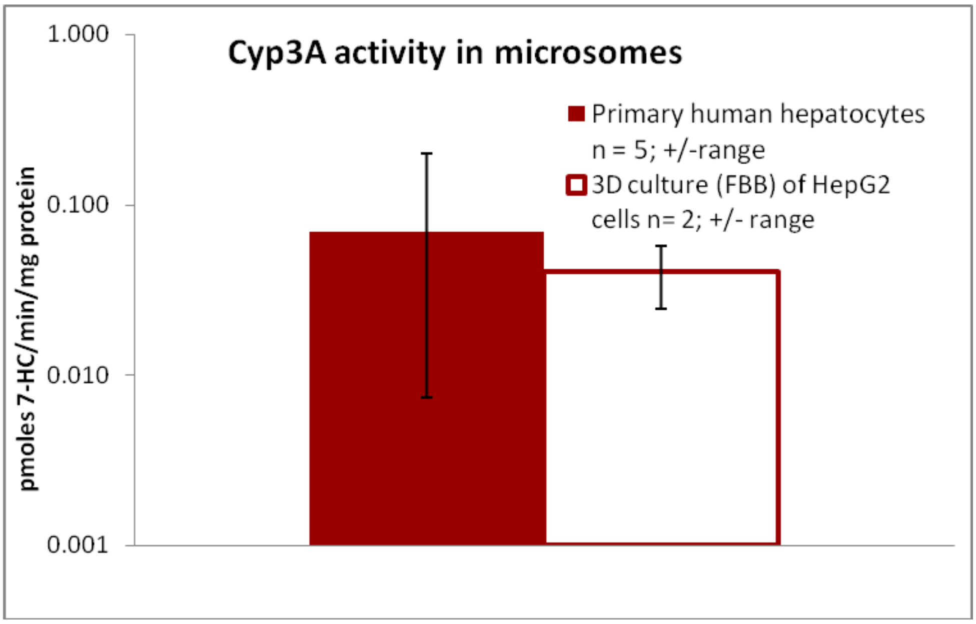

Figure 2 (supplementary). Cytochrome P450 activity in FBB biomass compared with hepatocytes from patients. *(a) ethoxycoumarin conversion in primary human hepatocytes and FBB biomass. 7-ethoxycoumarin (7-EC, 200um) was used as a substrate; 7-ethoxycoumarin-O-deethylation (ECOD) results in 7-hydroxycoumarin product formation by incubation for 1h at 37C and extraction of product. Fluorescence was determined in a Cytofluor microplate reader at excitation 364/40 and emission 460/40. Results were normalised to protein estimated by the BCA assay: this reaction may be catalysed by cytochrome P450 enzymes from CYP1, CYP2 and CYP3 families. (b) Specific activities of Cytochrome P4503A in microsomes isolated from primary human hepatocytes compared with FBB biomass culture. A Promega P450 GLO assay was used to determine isoform specific P450 3A*

	control-BAL	cell-BAL
ALK. PHOSPHATASE	340.0 ± 31.07 N=15	310.9 ± 33.06 N=13
AMMONIA PLASMA	348.5 ± 24.81 N=15	346.2 ± 37.99 N=13
ANION GAP	12.73 ± 1.217 N=15	13.31 ± 1.707 N=13
AST*	150.2 ± 26.04 N=16	311.4 ± 46.69 N=13
BICARBONATE	23.48 ± 1.441 N=15	24.15 ± 1.229 N=13
BILIRUBIN CONJUGATED	3.813 ± 0.3058 N=16	4.154 ± 0.4783 N=13
BILIRUBIN UNCONJUGATED	5.063 ± 0.5513 N=16	5.615 ± 0.6154 N=13
BILIRUBIN TOTAL	8.875 ± 0.7631 N=16	9.769 ± 0.9946 N=13
CREATININE	108.6 ± 4.387 N=16	100.1 ± 5.579 N=13
FIBRINOGEN	1.264 ± 0.1003 N=14	1.592 ± 0.2138 N=13
GLUCOSE	4.507 ± 1.135 N=14	4.391 ± 0.7204 N=11
HAEMOGLOBIN	11.31 ± 0.6563 N=15	11.73 ± 0.2986 N=13
INR	1.700 ± 0.5553 N=16	1.169 ± 0.04583 N=13
LACTATE	8.725 ± 1.051 N=16	8.023 ± 1.169 N=13
PATIENT PTT	95.68 ± 13.61 N=15	80.23 ± 12.47 N=13
PROTHROMBIN TIME	23.43 ± 10.45 N=16	13.32 ± 0.4791 N=13
WHITE CELL COUNT	14.83 ± 1.102 N=15	18.88 ± 2.225 N=13

Table 1 (supplementary) showing blood biochemistry analysed prior to BAL addition. *Parameters did not differ between pigs in control- and Cell-BAL groups, except AST, *p=0038.*

Parameters	Description of Parameters	Units	Control-BAL	Cell-BAL
APsys	Arterial systolic pressure	mmHg	106.8 ± 17.16 N=15	114.3 ± 24.71 N=12
APdia	Arterial diastolic pressure	mmHg	57.07 ± 12.14 N=15	63 ± 17.17 N=12
APmean	Mean Arterial pressure	mmHg	76.47 ± 13.96 N=15	83.08 ± 18.49 N=12
HR	Heart Rate	bpm	143.8 ± 32.84 N=15	134.1 ± 35.42 N=12
PCCO	Pulse Contour Cardiac Output	litres/min	3.991 ± 0.9786 N=14	4.142 ± 1.401 N=12
PCCI	Pulse Contour Cardiac Index	litres/min/m^2	4.596 ± 1.041 N=14	4.767 ± 1.537 N=12
SV	Stroke Volume	mls	28.64 ± 9.572 N=14	32.42 ± 11.93 N=12
SVI	Stroke Volume Index	Mls/m^2	32.64 ± 9.889 N=14	37.08 ± 12.83 N=12
SVmin	Stroke Volume minimum	mls	25.14 ± 8.787 N=14	29.33 ± 11.25 N=12
SVmax	Stroke Volume maximum	mls	30.07 ± 9.531 N=14	34.67 ± 12.45 N=12
SVV%	% Stroke Volume Variation	%	17.86 ± 5.869 N=14	17.42 ± 8.826 N=12
PPV%	% Pulse Pressure Variation	%	22.6 ± 7.595 N=15	21.83 ± 8.653 N=12
CVP	Central Venous Pressure	mmHg	9.4 ± 1.242 N=15	9.5 ± 1.168 N=12
SVR	Systemic Vascular Resistance	mmHg·min·mL^{-1}	1807 ± 1410 N=15	1524 ± 444.8 N=12
SVRI	Systemic Vascular Resistance Index	dyn*s*cm-5*m^2	1551 ± 1188 N=15	1314 ± 383.1 N=12
dPmx	Left Ventricular Contractility	mmHg/s	887.3 ± 367.2 N=15	899.2 ± 369.7 N=12
TBlood	Blood Temperature	degrees celsius	38.93 ± 1.469 N=15	39.04 ± 0.7902 N=12

Table 2 (supplementary) showing haemodynamic parameters analysed prior to BAL addition. *Parameters did not differ between pigs in control- and Cell-BAL groups*

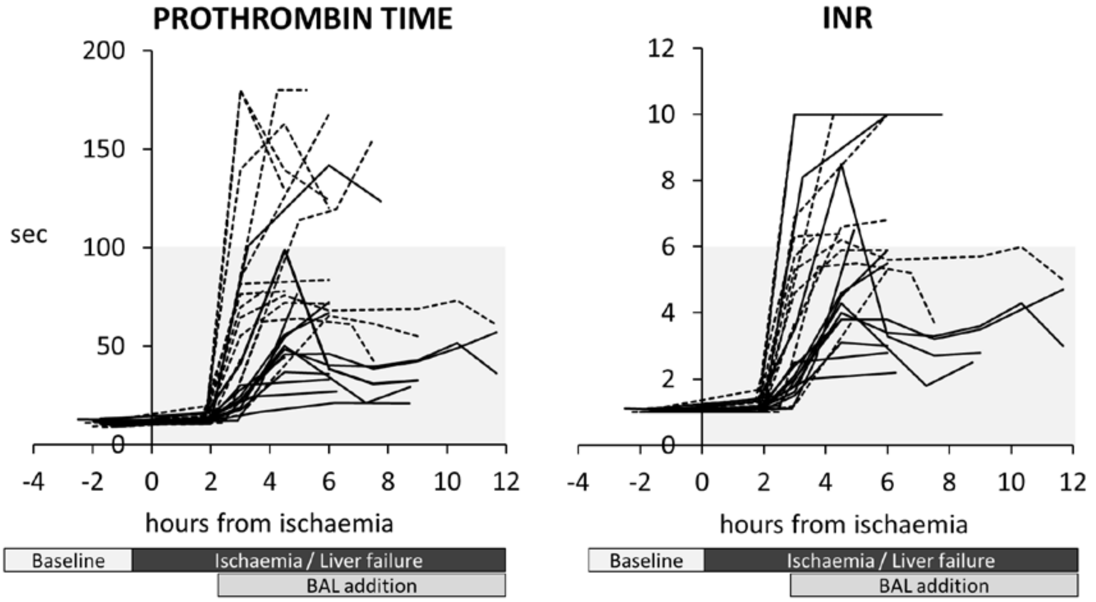

Figure 3 (supplementary): Prothrombin time and International Normalised Ratio (INR) in individual pigs. *Control-BAL: dashed line, Cell-BAL: solid line, BAL on at 2.80±0.5h from ischaemia. Prothrombin pig baseline = 11.0 ± 1 sec, INR pig baseline = 1.0 ± 0 (n=29, ±SD). Shaded area represents values above which a patient would be listed for urgent transplant; Prothrombin time >100s, INR >6.*

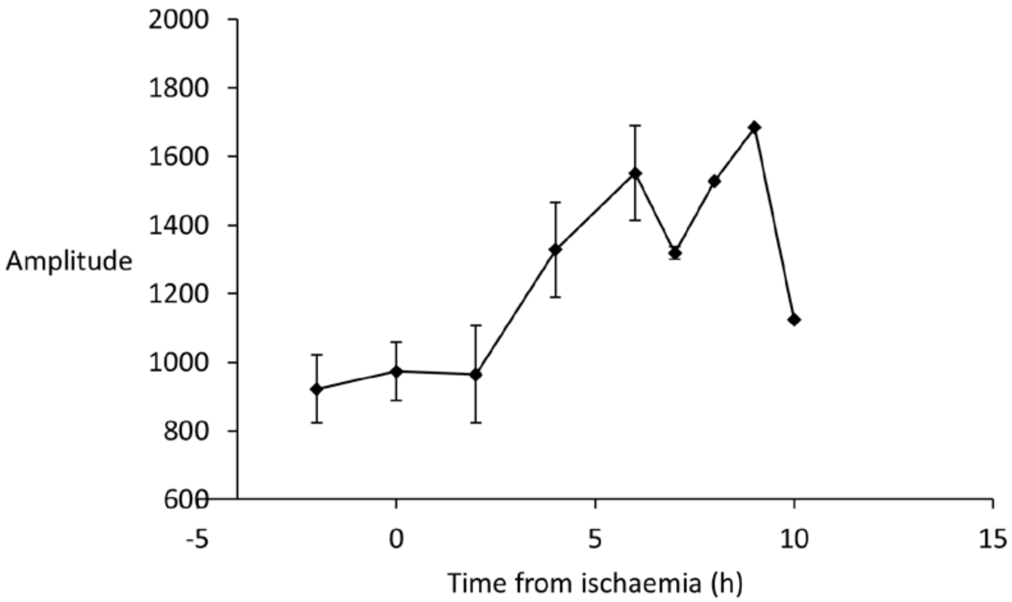

Figure 4 (supplementary). Rotem measurement of coagulation: fibtem*. Fibtem is a surrogate marker of Fibrinogen's contribution to coagulation and assessed by MCF representing the amplitude of the clot. Increase in the MCF amplitude indicates fibrinogen being produced by the BAL, enhancing clot strength. Mean +/- SEM, n=10; where error bars not present time points in individual pigs were not identical.*

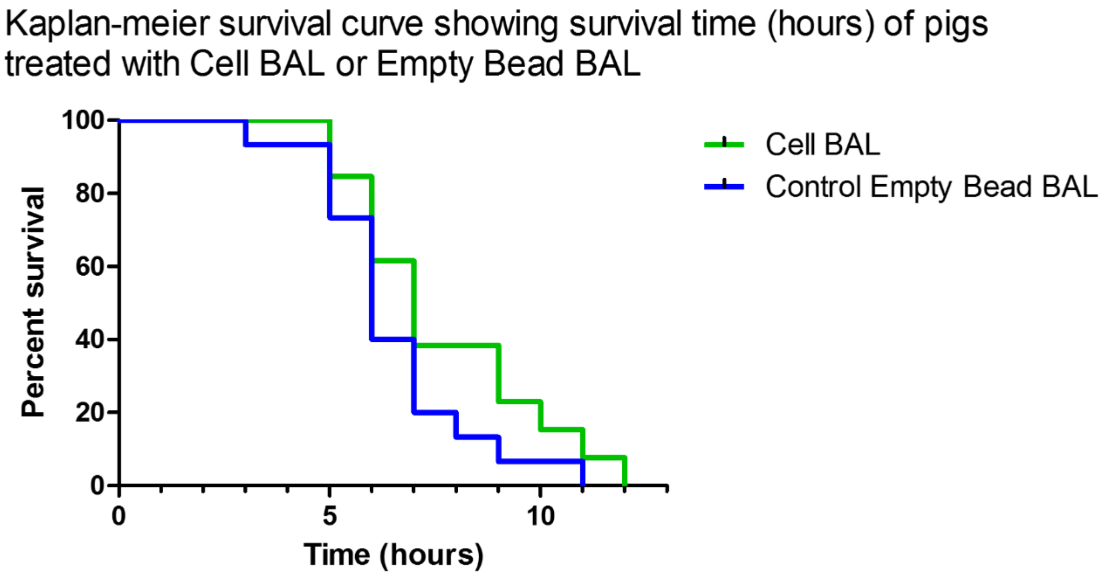

***Figure 5 (supplementary)**. Kaplan Meier survival curve: There was no significant difference in survival, although as alluded to in the manuscript there was a small increase in survival time observed. Time is shown as hours from ischaemia to death for all the animals in control treated (blue) and cell-treated (green) groups. This was expected in a permanent injury non survival model.*
